# In Vitro Antimycobacterial Activity and Physicochemical Characterization of Diaryl Ether Triclosan Analogues as Potential InhA Reductase Inhibitors

**DOI:** 10.3390/molecules25143125

**Published:** 2020-07-08

**Authors:** Tarek S. Ibrahim, Ehab S. Taher, Ebtihal Samir, Azizah M. Malebari, Ahdab N. Khayyat, Mamdouh F. A. Mohamed, Riham M. Bokhtia, Mohammed A. AlAwadh, Israa A. Seliem, Hani Z. Asfour, Nabil A. Alhakamy, Siva S. Panda, Amany M. M. AL-Mahmoudy

**Affiliations:** 1 Department of Pharmaceutical Chemistry, Faculty of Pharmacy, King Abdulaziz University, Jeddah 21589, Saudi Arabia; amelibary@kau.edu.sa (A.M.M.); ankhayyat@kau.edu.sa (A.N.K.); Malawadh@kau.edu.sa (M.A.A.); 2 Department of Pharmaceutical Organic Chemistry, Faculty of Pharmacy, Zagazig University, Zagazig 44519, Egypt; rbokhtia@augusta.edu (R.M.B.); isliem@augusta.edu (I.A.S.); amanysinger77@gmail.com (A.M.M.A.-M.); 3 Department of Pharmaceutical Organic Chemistry, Faculty of Pharmacy, Al-Azhar University, Assiut 71524, Egypt; ehaborganic@yahoo.com; 4 Physical Chemistry, Department of Analytical Chemistry, Faculty of Pharmacy, Deraya University, New Minia 61519, Egypt; Ebtihal_samir@yahoo.com; 5 Department of Pharmaceutical Chemistry, Faculty of Pharmacy, Sohag University, Sohag 82524, Egypt; mamdouhfawzy3@yahoo.com; 6 Department of Chemistry & Physics, Augusta University, Augusta, GA 30912, USA; 7 Department of Medical Microbiology and Parasitology, Faculty of Medicine, King Abdulaziz University, Jeddah 21589, Saudi Arabia; hasfour@kau.edu.sa; 8 Department of Pharmaceutics, Faculty of Pharmacy, King Abdulaziz University, Jeddah 21589, Saudi Arabia; nalhakamy@kau.edu.sa

**Keywords:** diphenyl ether, H37Rv, antitubercular agents, InhA inhibitors, molecular docking

## Abstract

Two sets of diphenyl ether derivatives incorporating five-membered 1,3,4-oxadiazoles, and their open-chain aryl hydrazone analogs were synthesized in good yields. Most of the synthesized compounds showed promising in vitro antimycobacterial activity against *Mycobacterium tuberculosis* H37Rv. Three diphenyl ether derivatives, namely hydrazide **3**, oxadiazole **4** and naphthylarylidene **8g** exhibited pronounced activity with minimum inhibitory concentrations (MICs) of 0.61, 0.86 and 0.99 μg/mL, respectively compared to triclosan (10 μg/mL) and isoniazid (INH) (0.2 μg/mL). Compounds **3**, **4**, and **8g** showed the InhA reductase enzyme inhibition with higher IC_50_ values (3.28–4.23 µM) in comparison to triclosan (1.10 µM). Correlation between calculated physicochemical parameters and biological activity has been discussed which justifies a strong correlation with respect to the inhibition of InhA reductase enzyme. Molecular modeling and drug-likeness studies showed good agreement with the obtained biological evaluation. The structural and experimental information concerning these three InhA inhibitors will likely contribute to the lead optimization of new antibiotics for *M. tuberculosis*.

## 1. Introduction

**Figure 1:**
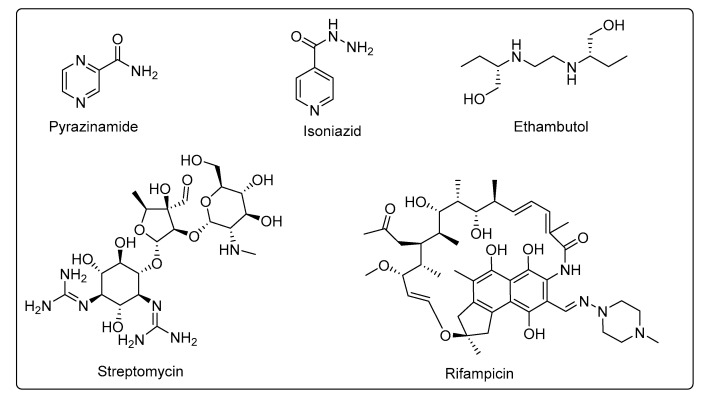
Commercially available anti-tubercular drugs.

Tuberculosis (TB), caused mainly by *Mycobacterium tuberculosis* (MTB), remains the world’s deadliest infection, having recently surpassed HIV as the leading cause of infectious disease mortality. The risk of developing tuberculosis (TB) is estimated as 16–27 times greater in people living with HIV than among those without HIV infection [1]. About 4500 people lose their lives, and close to 30,000 people fall ill each day with this preventable and curable disease [2]. The treatment plan for MTB infection is strenuous, requires long-term treatment of combination chemotherapy of two or more drugs, like pyrazinamide, isoniazid (INH), rifampicin, streptomycin and/or ethambutol (Figure 1). This combination usually accompanied by poor patient compliance, which leads to emerging of multi-drug resistant (MDR-TB) strains [3].

**Figure 2:**
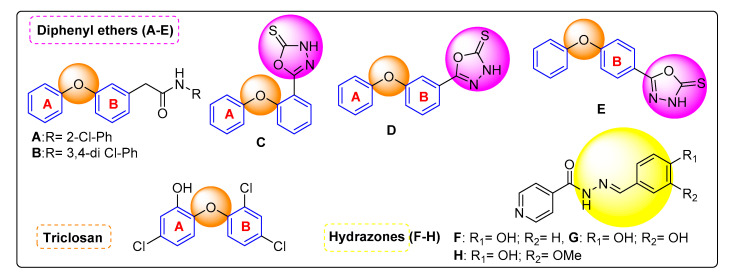
Structures of triclosan, diphenyl ether, and hydrazone derivatives.

Although one possible long-term solution to this issue is a better vaccine, in the near future, the major reliance will be on chemotherapy requiring an urgent development of potential, non-toxic, and effective antitubercular agents against latent MTB. In the search for novel TB lead candidates, the enoyl-acyl carrier protein, InhA reductase has become a target of interest by pharmaceutical companies and academic institutions for the development of new anti-tubercular drugs. InhA is the key enzyme that catalyzes NADH-dependent reduction of 2-trans-enoyl-ACP (acyl carrier protein) to give up NAD^+^ and reduced enoyl thioester-ACP substrate, which in turn, aids in the synthesis of mycolic acid. Mycolic acid is a unique signature fatty acid and considered as a core constituent of the mycobacterial cell wall [4]. InhA enzyme has been identified as the key target of the most currently existing anti-TB drugs isoniazid (INH), and ethionamide. Most cases of resistance to these drugs result from mutations in the mycobacterial activating enzyme (catalase-peroxidase KatG for isoniazid and EthA for ethionamide) [5,6,7,8,9,10,11,12,13]. Also, triclosan (Figure 2), the non-ionic lipophilic anti-TB agent, has been identified as a promising reversible inhibitor of InhA. This diphenyl ether molecule doesn’t require any bioactivation and capable of directly inhibit InhA. This approach probably allows avoiding the dominant cause of the resistance to isoniazid consisting of KatG mutations. SAR studies of triclosan revealed that chlorine atoms in ring B are not significant in the activity while ring B itself is essential moiety for inhibition of InhA (Figure 2); it makes several hydrophobic contacts with adenoyl acyl carrier protein reductase enzyme. The ether oxygen bond is also crucial for the activity due to two reasons. Initially, it is necessary for maximizing the interaction of triclosan with InhA and consequently better biological activity [14]. Secondly, being of critical in the formation of the stable ternary InhA-triclosan-NAD^+^ complex. Replacement of this group by a sulfur atom abolishes the inhibitory activity [13,15]. Different literature studies have been carried out based on the above hypothesis. Yang and coworkers carried out several of Des-chloro-diphenyl ether derivatives such as A and B (Figure 2), in the absence of the phenolic group, to enhance pharmacokinetic and pharmacodynamic properties thereby improving the druggability of triclosan. These compounds showed good antitubercular potency against MTB strain H37Rv (Figure 2) [16].

On the other facet, oxadiazoles represent an important class of 5-membered heterocyclic systems that cover a broad range of biological activities such as antimycobacterial [17], pesticide [18], anti-inflammatory and analgesic [19], antimicrobial [20] and antitumor [21,22], In the search of more effective anti-tuberculosis, oxadiazole is one of the most versatile scaffolds against MTB strain. Interestingly, extensive chemical and structural investigations have been performed by Kini and his group through developing series of oxadiazole *o*/*m*/*p*-substituted diphenyl ether derivatives (C–E) which displayed inhibition against the growth of the H37Rv strain of mycobacterium (Figure 2) [9]. The hydrazide hydrazone skeleton is also a class of privileged structures with a broad range of biological activities such as antioxidant [23], anti-inflammatory [24], and antitubercular activities [25,26]. Recently, in a high throughput screening for inhibitors of *M. tuberculosis* H37Rv, several aryl hydrazine derivatives (F–H, Figure 2) [27] were found to possess potent activity in combination with the relatively low toxicity [27]. However, despite the promising in vitro activity of the currently existing diphenyl ethers have poor bioavailability [28] and limited aqueous solubility [28], which is likely one reason why they have limited in vivo efficacy [10].

In continuation of our previous work in parallel to the above findings and the observed relationship between lipophilicity and in vivo efficacy, especially as it pertains to antimicrobial agents [5,6,7,8,9,10,11,12], new diphenyl ether analogs have been designed as potential antitubercular inhibitors of InhA reductase enzyme. This approach developed by keeping the known essential functional groups for the activity. Modification has been made to the diphenyl ether ‘B’ ring of diphenyl ether through binding the B ring with five-membered oxadiazole moieties either substituted or non-substituted ones; this might enhance bioactivity, pharmacokinetics or pharmacodynamics properties and could be a good lead for the generation of InhA reductase inhibitors. Furthermore, the substitution of the *para* position of diphenyl ether ‘B’ ring with aryl hydrazine or arylidene moiety as an open-chain analog of oxadiazole may improve solubility and enhance the antimycobacterial activity. Prediction of correlation between antitubercular activity with both Clog P and IC_50_ of InhA inhibition has been represented. Also, the correlation of the anti-TB activity with different thermodynamic, topological parameters was also considered. Molecular modeling studies were carried out to better understand the observed activity and determine the most important structural parameters participating in binding with InhA reductase that may affect the biological activity.

## 2. Results and Discussion

### 2.1. Chemistry

**Scheme 1:**
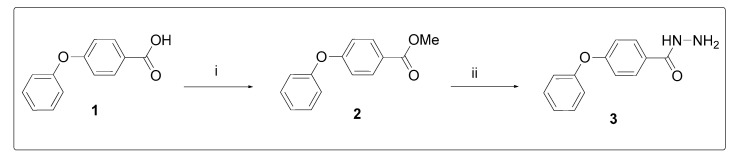
Reagents and conditions: (i) Conc.H_2_SO_4_, MeOH, reflux, 8 h; (ii) NH_2_NH_2_.H_2_O, EtOH, reflux, 3 h.

**Scheme 2:**
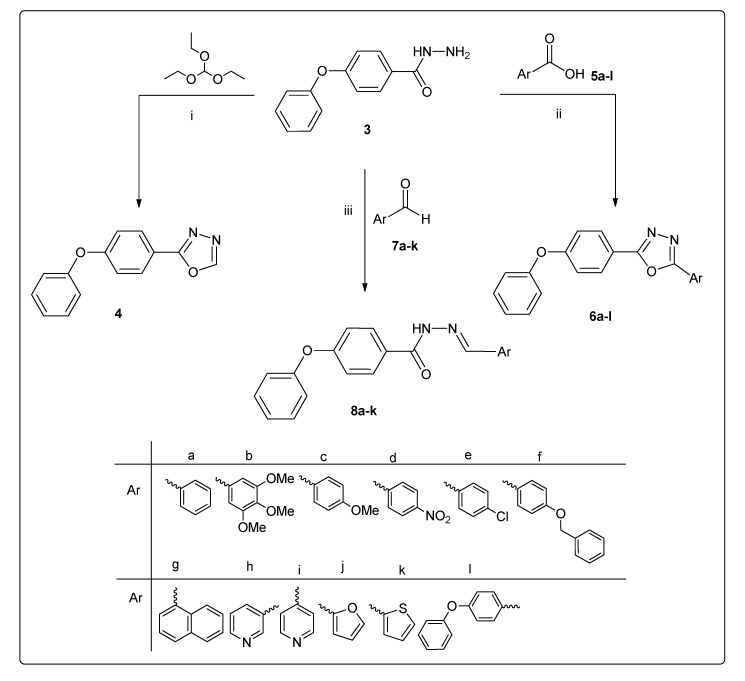
Reagents and conditions: (i) Triethyl orthoformate reflux, 3 h; (ii) POCl_3_, reflux, 2–3 h; (iii) EtOH, reflux, 3–6 h.

The target derivatives were prepared through the conversion of the starting phenoxybenzoic acid **1** into the corresponding ester **2** using methanol and concentrated sulphuric acid under reflux conditions [28,29,30]. Hydrazinolysis of the ester **2** with hydrazine hydrate gives the hydrazide **3** (Scheme 1). Cyclocondensation of hydrazide **3** through its reaction with triethyl orthoformate under refluxing conditions led to the formation of the oxadiazole **4** (Scheme 2). The reaction of the hydrazide **3** with the appropriate aromatic carboxylic acid derivatives **5a**–**l** in the presence of phosphorus oxychloride afforded the corresponding 5-aryl-substituted oxadiazoles **6a**–**l**. The condensation reaction of hydrazide **3** with variant aromatic aldehydes **7a**–**k** resulted in the formation of anticipated arylidenes **8a**–**k** (Scheme 2). The prepared target compounds were characterized by spectral studies.

### 2.2. Biology

#### 2.2.1. Anti-Tubercular Activity

Compounds **3**, **4**, **6a**–**l** and **8a**–**k** were evaluated for antitubercular activity against *Mycobacterium tuberculosis* H37Rv using the standard Microplate Alamar Blue Assay (MABA) protocol (Table 1) [31,32]. The MIC reported as the lowest concentration (μg/mL) of targets inhibited the growth of an organism. Triclosan and INH were used as reference standards. Among the tested compounds, the results displayed that most of them i.e., **3**, **4**, **6h**, **6i**, **6l**, **8g**, and **8k** showed promising anti-tubercular activity (MIC, 0.61–1.69 μg/mL) in comparison to triclosan (MIC, 10 μg/mL) and INH (MIC, 0.2 μg/mL). 

The structure-activity relationship acquired suggested that installing the open-chain oxadiazole moiety into the diphenyl ether caused a remarkable increase in activity as seen in compound **3** (MIC, 0.86 μg/mL). Interestingly, the cyclic and unsubstituted oxadiazole **4** (MIC, 0.61 μg/mL) showed experienced and superior activity than all the 5-substituted analogs. Noteworthy, the 5-phenyl **6a** and 5-benzyloxy-phenyl **6f** derivatives experienced a slight decrease in activity with MIC of 2.74, 8.23 μg/mL, respectively. It was noted, however, that replacement of the phenyl group at the oxadiazole ring with a six-membered heterocyclic system showed a slight improvement in the activity as depicted in compounds **6h** and **6i** (MIC, 1.40 μg/mL) while alteration of this ring into 5-membered one exhibited less activity such as **6k** (MIC, 3.65 μg/mL). Expectedly, bis(diphenylether) oxadiazole **6l** was the most active in this 5-substituted series (1.17 μg/mL) that may be due to the incorporation of extra phenyl ether nucleus to the backbone. The substitutions on the 5-aryl groups have a deleterious effect on biological activity especially the activating methoxy substituents in compounds **6b** and **6c**.

In a related vein, the arylidene group of compounds **8a**–**k** was found comparably less active in vitro where this situation may modify in vivo, as we know arylidenes are prodrugs. By comparing these derivatives, it is clear that the introduction of the heterocyclic system into the diphenyl ether hydrazone structure either five, compound **8j** (MIC, 6.35 μg/mL) or six-membered ring system, compounds **8h**–**i** (MIC ~ 6.90 μg/mL) caused a decrease in the anti-tubercular activity. Similarly, replacement of the heterocyclic ring with substituted phenyl group i.e., compounds **8d** (MIC, 7.67 μg/mL) and **8e** (MIC, 2.89 μg/mL) showed weak inhibition. Surprisingly, a remarkable increase in the activity was observed for compounds **8g** (0.98 μg/mL; Ar = Napthyl) and **8k** (MIC, 1.69 μg/mL; Ar = thienyl). Generally, the order of activity against MTB strain H37Rv of diphenyl ether bearing arylidene core was Ar = zero > naphthyl > thienyl > *p*-Cl-phenyl > 3 pyridinyl ~ 4-pyridinyl > *p*-NO_2_-phenyl. All these data suggested that the diaryl ether scaffold is very important for the antitubercular activity, removal of the *o*-chloro group in triclosan, as well as the introduction of oxadiazole or its open-chain analog hydrazide, increased activity than the parent triclosan. In conclusion, compounds **3**, **4**, **6h**, **6i**, **6l**, **8g**, and **8k** showed higher antitubercular activity than the reference standard triclosan. Three potential lead candidates **3** and **4** and **8g** were found to be the most active in which hydrazide oxadiazole or naphthyl arylidine moieties are directly attached to an unsubstituted diphenyl ether core. Their structural features could be further investigated for their potential use as new anti-TB drugs.

#### 2.2.2. In Vitro InhA Reductase Enzyme Inhibition 

The in vitro inhibitions data of the target compounds for the InhA reductase enzyme are presented as IC_50_ µM values in Table 1. Among the tested compounds, the highly active hydrazide **3** and oxadiazole **4** and naphthyl arylidine **8g** showed the highest inhibition of InhA reductase with IC_50_ of 3.43 µM and 3.28 µM and 4.23 µM, respectively, but still less than the reference triclosan (IC_50_, 1.10 µM). On the other hand, oxadiazole derivatives **6a**–**c** and the corresponding arylidenes **8a**–**c** showed deleterious activity as inhibitors for InhA reductase. The oxadiazoles **6h**, **61** and arylidine **8k** ether derivatives experienced good inhibition activities with IC_50_ of 5.08, 5.63, and 5.61, respectively. In general, most of the tested target compounds showed good inhibition for InhA reductase. Besides, other compounds show promising activity against H37Rv strain of *M. tuberculosis*. We believe, additional modes of mechanisms involved in the activity. 

Moreover, calculated results showed a slight correlation between the molecular weight, Wiener index, and topological diameter to biological activity with R = 0.308, 0.315, 0.259, respectively. These results indicate the importance of the overall volume of a molecule on the biological activity, but it is not the only factor affecting biological activity. On the other hand, it was found that all the thermodynamic parameters (i.e., molar refractivity and Gibbs free energy and heat formation) have a negative correlation with the biological activity where R = −0.100, −0.602 and −0.683, respectively (Table 2).

The molecules that display a negative sign for the heat of formation parameter means that binding of these molecules with their protein targets may be spontaneous and do not require energy. The protein-ligand binding occurs only when the change in Gibbs free energy (Δ*G*: is the free energy that gives an indication about a binding affinity to the target site) of the system is negative. Furthermore, Hydrogen bonding is one of the important factors which is responsible for determining the three-dimensional structure of folded proteins including enzymes and the ligand molecule. Also, it is involved in determining the polarity and permeability of compounds. Calculations showed that antimycobacterial activity expressed in MIC (μg/mL) is negatively correlated with both hydrogen bond donor and hydrogen bond acceptor count. Calculation of the number of hydrogen bond donors, hydrogen bond acceptors, and other parameters of Lipinski’s (rule of five) showed that most of the target compounds presented drug-like characters [33,34,35].

#### 2.2.3. Cytotoxic Study

Compounds **3**, **4**, **6h**, **6i**, **6l**, **8g**, and **8k** displaying the lowest MIC values were selected for cytotoxicity test using model eukaryotic cell line (Vero) at the concentration range from 0.1 to 200 μg/mL with IC_50_ values more than 60 μg/mL. Compounds did not show a high cytotoxic effect on Vero cells (Table 3). 

Notably, compound **4** which showed the lowest MIC value of 0.61 μg/mL also displayed very low cytotoxicity to Vero cells at all tested concentrations (0.1–200 μg/mL) with IC_50_ values close to 100 µg/mL (99.4) and accordingly its selectivity index (SI) value more than 10 (162.95), providing its promising potential in biomedical application. In the case of compounds **3**, **6h**, **6i**, and **6l**, a slight reduction in cell viability was observed with IC_50_ ranging from 80.7–95.5 µg/mL, whereas **8g** and **8k** showed the more pronounced cytotoxic effect was with IC_50_ 72.3 and 68.6 µg/mL, respectively. Therefore, it may be assumed that **4**, **6h**, **6i**, **6l**, **8g**, and **8k** derivatives, which were non-toxic or showed very low toxicity at tested concentration range, are very promising and potentially safe antimycobacterial compounds.

#### 2.2.4. Molecular Docking

**Figure 3:**
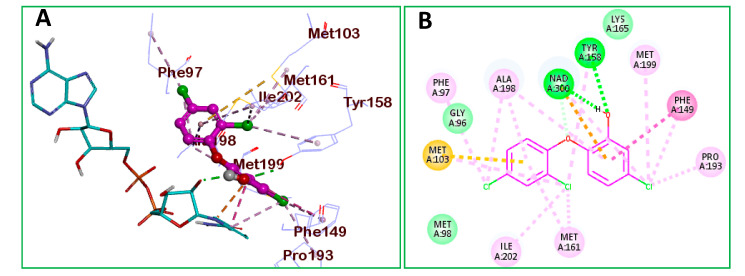
Docking and binding mode of Triclosan into the active site of InhA reductase enzyme (PDB code: 3FNH). (**A**) 3D structure of triclosan (pink), (**B**) 2D structure of Triclosan (pink).

The inhibitory profile of triclosan and compounds **3**, **8g** (models for the higher inhibitory activity) and **8b** (model for the deleterious inhibitory activity) were chosen for further investigation by docking into InhA reductase active site (PDB code: 3FNH) to gain insight into the potential binding modes [36,37,38]. As a first step, for the validation of docking parameters, the co-crystal ligand (JPJ400) was re-docked at the catalytic site of the protein. The RMSD between co-crystal and re-docked poses was found to be 0.331 (<2A), which confirmed the validity of the docking parameters. Triclosan exploited two major interactions. Firstly, the hydrophilic interaction has been noticed by the hydroxyl OH via two hydrogen bonds i.e., the oxygen engaged with the hydrophilic side chainTyr158 while the hydrogen forms the second hydrogen bond with NAD. Secondly, clusters of hydrophobic interactions including pi—or thirteen—alkyl interactions with NAD, Phe97, Met161, Pro193, Ala198, Met199, and Ile202 were also found. Further, pi-cation interaction with NAD, pi-sulfur interaction with Met103 and also, pi-pi stacked interaction with Phe149 have been noticed as well (Figure 3).

**Figure 4:**
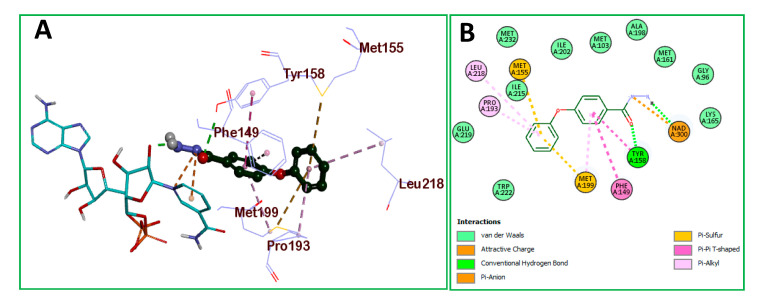
Docking and binding mode of compound **3** into the active site of InhA reductase enzyme (PDB code: 3FNH). (**A**) 3D structure of compound **3** (green), (**B**) 2D structure of compound **3** (green).

**Figure 5:**
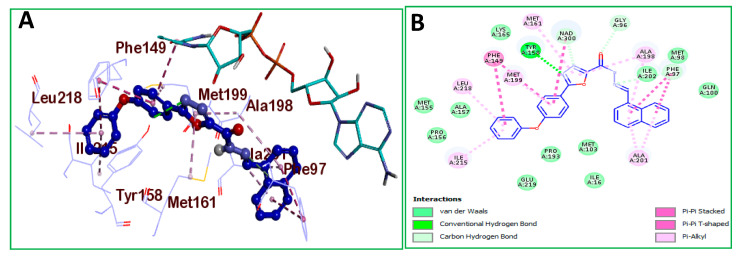
Docking and binding mode of compound **8g** into the active site of InhA reductase enzyme (PDB code: 3FNH). (**A**) 3D structure of compound **8g** (blue), (**B**) 2D structure of compound **8g** (blue).

By careful analysis of the docked poses of compounds **3** and **8g**, it was clear that both compounds can occupy the same binding mode as Triclosan with binding interaction energy (−24.264) kcal/mol (for compound 3) to −17.148 kcal/mol (for compound **8g**) compared to Triclosan (−22.507) kcal/mol. The hydrophilic interaction has been noticed in compound **3** via the carbonyl oxygen that forms a hydrogen bond with Tyr158 side chain, while the hydrogen of amino group forms the second one with NAD, but compound **8g** was involved in only one i.e., N4 of the oxadiazole ring forms one hydrogen bond with the hydrophilic residue Tyr 158, likely due to its bulkiness and this may explain its low potency than compound **3** (Figure 4 and Figure 5). The hydrophobic interactions in compound **3** were incorporating three pi-alkyl interactions with Pro193, Met199 and Leu218. Moreover, two pi-sulfur interactions with Met155 and Met199, one attractive charge interaction with NAD and two pi-pi T-shaped interactions with Phe149 and Tyr158 have been observed. In the same mode, compound **8g** displays its hydrophobic interactions with different amino acid residues that embodying five pi-pi stacked interactions with NAD, Phe97, and Phe149. Also, eight pi-alkyl interactions with Phe149, Met161, Ala198, Ala201, Ile215, and Leu218 were found.

**Figure 6:**
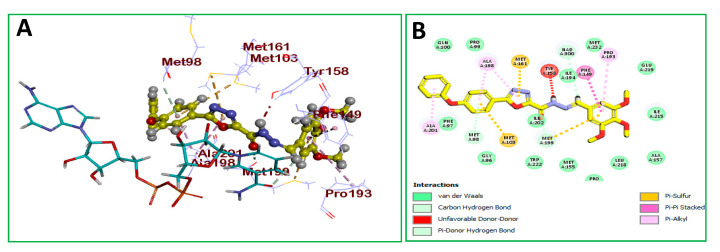
Docking and binding mode of compound **8b** into the active site of the InhA reductase enzyme (PDB code: 3FNH). (**A**) 3D structure of compound **8b** (yellow), (**B**) 2D structure of compound **8b** (yellow).

It is worth pointing out that in compound **8b**, the hydrogen of the hydrazide group showed unfavorable donor-donor interaction (red color) with binding interaction energy (+11.145) kcal/mol. Also, the compound has not involved in any hydrogen bonds and forms only hydrophobic interaction with some amino acid residues and this may reflect the low activity of the compound (Figure 6).

## 3. Material and Methods

### 3.1. Chemistry

Melting points were recorded on a Stuart SMP30 (Stuart, Cole-Parmer, Staffordshire, UK) melting point apparatus. IR spectra (KBr, cm^−1^) were recorded on a Vector 22FT-IR Fourier Transform infrared (FTIR) spectrometer (Bruker, Billerica, MA, USA). ^1^H-NMR and ^13^C-NMR spectra were recorded at 500 and 125 MHz, respectively, on an Inova 500 MHz spectrometer (Bruker, Billerica, MA, USA), using dimethyl sulphoxide (DMSO-d_6_)/deuterated chloroform (CDCl_3_) as a solvent and tetramethylsilane (TMS) as an internal standard (chemical shift in δ, ppm). Mass spectra were determined using a Shimadzu GC/MS QP 1000 EX system (Shimadzu Corporation, Tokyo, Japan) with ionization energy 70 eV. Elemental analysis were determined using an Automatic Elemental Analyzer CHN Model 2400 (Perkin Elmer, Richmond, CA, USA) at the Micro-analytical Center, Faculty of Science, Cairo University (Cairo, Egypt). All the results of elemental analyses corresponded to the calculated values within experimental error. Progress of the reaction was monitored by thin-layer chromatography (TLC) using precoated TLC sheets with ultraviolet (UV) fluorescent silica gel (60F254, Merck, Kenilworth, NJ, USA) and spots were visualized by iodine vapors or irradiation with UV light (254 nm). All the chemicals were purchased from Sigma-Aldrich Chemicals, St. Louis, MO, USA. Compounds **2**, **3**, **4**, **6g**, **6i**–**k** and **8i**–**k** were prepared according to the reported procedures [30,39,40].

*Methyl 4-phenoxybenzoate* (**2**). Concentrated H_2_SO_4_ (1.0 mL) was added dropwise to a solution of 4-phenoxybenzoic acid (3.3 g, 15.6 mmol) in methanol (25 mL). The reaction mixture was allowed to reflux for 8 h. The reaction mixture was concentrated under reduced pressure. The obtained residue was treated with 10% aqueous NaHCO_3_ (10 mL). Stirring was continued for 15 m. During which time, the precipitate was formed then collected by filtration then dried and purified by crystallization using methanol to afford white solid in 80% yield; m.p. 159–160 °C; ^1^H-NMR (CDCl_3_) δ: 3.90 (s, 3H, OCH_3_), 6.99 (d, *J* = 8.8 Hz, 2H, ArH), 7.07 (d, *J* = 7.8 Hz, 2H, ArH), 7.19 (t, *J* = 7.9 Hz, 1H, ArH), 7.39 (t, 2H, *J* = 7.9 Hz, ArH), 8.00 (d, *J* = 8.4 Hz, 2H, ArH); ^13^C-NMR (CDCl_3_) δ: 52.1 (OCH_3_), 117.4(C3, C5), 120.2 (C3′, C5′), 124.6 (C1), 130.2 (C2′, C6′), 131.8 (C2, C6), 155.8 (C4), 162.0 (C1′), 166.7 (C4′), 207.1 (C=O).

*4-Phenoxybenzohydrazide* (**3**). Methyl 4-phenoxybenzoate (228 mg, 1 mmol) was added to a solution of hydrazine hydrate 98% (0.5 mL) in ethanol (5 mL). The mixture was refluxed for 2 h. The reaction mixture was evaporated under vacuum, water (10 mL) was added to the obtained residue. The obtained mixture was extracted with ethyl acetate (2 × 10 mL). The combined organic layers were then washed with a saturated solution of sodium bicarbonate and (1 × 10 mL) then brine (1 × 10 mL) before being dried (MgSO_4_). The obtained filtrate was concentrated under reduced pressure, filtered off and the precipitate was subjected to crystallization using EtOAc/petroleum ether. White solid, Yield: 84%; m.p.: 180–190 °C; ^1^H-NMR (CDCl_3_) δ: 4.49 (s, 2H, NH_2_), 7.00 (d, *J* = 8.0 Hz, 2H, ArH), 7.05 (d, *J* = 8.0 Hz, 2H, ArH), 7.18 (t, *J* = 7.5 Hz, 1H, ArH), 7.36–7.40 (m, 2H, ArH), 7.41 (s, 1H, NH), 7.72 (d, *J* = 8.2 Hz, 2H, ArH). ^13^C-NMR (CDCl_3_) δ: 118.0 (C3, C5), 120.1 (C3′, C5′), 124.6 (C1), 127.1 (C1′), 128.9 (C2′, C6′), 130.2 (C2, C6), 155.9 (C4), 161.1 (C4′), 168.3 (C=O).

*2-(4-Phenoxyphenyl)-1,3,4-oxadiazole* (**4**). A mixture of hydrazide **3** (1 mmol) and triethyl orthoformate (2 mL) were refluxed for 3 h. The reaction mixture was concentrated under vacuum; the residue was cooled and then treated with hexane (5 mL). The obtained precipitate was filtered off and recrystallized from ethanol/water (1:1). White solid, Yield: 73%; m.p. 140–144 °C [26]. ^1^H-NMR (DMSO-*d*_6_) δ: 7.14–7.17 (m, 4H, ArH), 7.25 (t, *J* = 7.5 Hz, 1H ArH), 7.45–7.49 (m, 2H, ArH), 8.01–8.04 (m, 2H, ArH), 9.31 (s, 1H, CH-oxadiazole). ^13^C-NMR (DMSO-*d*_6_) δ: 117.8 (C3, C5), 118.3 (C3′, C5′), 119.9 (C1), 124.7 (C1′), 128.9 (C2, C6), 130.3 (C2′, C6′), 154.2 (C4′), 155.1 (C4), 160.2 (C=N), 163.3 (C=N). Anal. Calcd. for C_14_H_10_N_2_O_2_: C, 70.58; H, 4.23; N, 11.76. Found: C, 70.50; H, 4.48; N 11.37.

*2-(4-Phenoxyphenyl)-5-phenyl-1,3,4-oxadiazole* (**6a**). White solid, Yield; 70%; m.p. 233–235 °C. ^1^H-NMR (CDCl_3_) δ: 7.01–7.07 (m, 5H, ArH), 7.18–7.22 (m, 1H, ArH), 7.38–7.56 (m, 5H, ArH), 7.85–7.88 (m, 3H, ArH). ^13^C-NMR (CDCl_3_) δ: 118.0 (C3, C5), 119.1 (C3′, C5′), 120.3 (C3″), 122.9 (C5″), 124.8 (C1), 125.7 (C1′, C1″), 127.5 (C2, C6), 129.1 (C2′, C6′), 129.5 (C2″), 130.3 (C6″), 131.6 (C4), 132.7 (C4′), 161.7, (C4″), 164.3 (C=N), 164.5 (C=N). Anal. Calcd for C_20_H_14_N_2_O_2_: C, 76.42; H, 4.49; N, 8.91. Found: C, 76.54; H, 4.23; N 9.27. 

*2-(4-Phenoxyphenyl)-5-(3,4,5-trimethoxyphenyl)-1,3,4-oxadiazole* (**6b**). White solid, Yield; 76%, m.p. 250–253 °C. ^1^H-NMR (CDCl_3_) δ: 3.92 (s, 3H, OMe), 3.97 (s, 6H, 2OMe), 7.05 (d, *J* = 8.0 Hz, 2H, ArH), 7.18–7.24 (m, 3H, ArH), 7.34 (s, 2H, ArH), 7.64–7.70 (m, 2H, ArH), 7.98 (d, *J* = 8.2 Hz, 2H, ArH). ^13^C-NMR (CDCl_3_) δ: 56.7 (OMe), 56.8 (OMe), 61.3 (OMe), 104.4 (C3, C5), 104.7 (C3′, C5′), 118.5 (C3″, C5″), 119.2 (C1, C1′), 120.2 (C1″), 124.8 (C2, C6), 129.1 (C2′, C6′), 130.3 (C2″, C6″), 141.4 (C4), 153.9 (C4′), 155.9 (C4″), 161.0 (C=N), 162.4 (C=N). Anal. Calcd for C_23_H_20_N_2_O_5_: C, 68.31; H, 4.98; N 6.93. Found: C, 67.92; H, 5.02; N 7.05. 

*2-(4-Methoxyphenyl)-5-(4-phenoxyphenyl)-1,3,4-oxadiazole* (**6c**). White solid, Yield; 72%, m.p.: 226–228 °C. ^1^H-NMR (CDCl_3_) δ: 3.87 (s, 3H, OMe), 7.00–7.02 (m, 2H, ArH), 7.10–7.20 (m, 4H, ArH), 7.17–7.20 (m, 1H, ArH), 7.37–7.40 (m, 2H, ArH), 8.03–8.07 (m, 4H, ArH). ^13^C-NMR (CDCl_3_,) δ: 55.7 (OMe), 114.8 (C3, C5), 116.7 (C3′, C5′), 118.5 (C3″, C5″), 118.7 (C1), 120.2 (C1′), 124.7 (C2, C6), 128.89 (C2′, C6′), 128.93 (C2″), 130.27 (C6″), 156.0 (C4), 160.9 (C4′), 162.6 (C4″), 1624.0 (C=N), 166.6 (C=N). Anal. Calcd for C_21_H_16_N_2_O_3_: C, 73.24; H, 4.68; N 8.13. Found: C, 73.62; H, 4.93; N 8.06. 

*2-(4-Nitrophenyl)-5-(4-phenoxyphenyl)-1,3,4-oxadiazole* (**6d**). White solid, Yield; 77%, m.p.: 226–228°C. ^1^H-NMR (CDCl_3_) δ: 7.09–7.13 (m, 4H, ArH), 7.19–7.24 (m, 1H, ArH), 7.42 (dd, *J* = 15.8, 8.0 Hz, 2H, ArH), 7.42 (dd, *J* = 16.0, 8.0 Hz, 2H, ArH), 8.28–8.45 (m, 4H, ArH). ^13^C-NMR (CDCl_3_) δ: 118.5 (C3), 120.2 (C5), 120.4 (C3′), 124.7 (C5′), 124.8 (C3″), 125.0 (C5″), 128.0 (C1), 129.0 (C1′), 129.3 (C1″), 129.7 (C2), 130.3 (C6), 130.4 (C2′, C6′), 149.7 (C2″, C6″) 155.6 (C4), 155.9 (C4′), 161.0 (C4″), 161.6 (C=N), 162.8 (C=N). Anal. Calcd for C_20_H_13_N_3_O_4_: C, 66.85; H, 3.65; N, 11.69. Found: C, 66.73; H, 3.39; N 11.92. 

*2-(4-Chlorophenyl)-5-(4-phenoxyphenyl)-1,3,4-oxadiazole* (**6e**). White solid, Yield; 72%, m.p.: 212–214 °C. ^1^H-NMR (CDCl_3_) δ: 7.09–7.11 (m, 4H, ArH), 7.20–7.21 (m, 1H, ArH), 7.40–7.43 (m, 2H, ArH), 7.51–7.53 (m, 3H, ArH), 8.06–8.09 (m, 4H, ArH). ^13^C-NMR (CDCl_3_) δ: 118.5 (C3), 120.2 (C5), 120.3 (C3′), 122.4 (C5′), 122.7 (C3″), 124.7 (C5″) 124.8 (C1), 128.4 (C1′), 128.5 (C1″), 129.0 (C2), 129.1 (C6), 129.7 (C2′), 129.8 (C6′), 130.28 (C2″), 130.30 (C6″), 138.2 (C4), 138.4 (C4′), 155.8 (C4″), 161.2 (C=N), 164.2 (C=N). Anal. Calcd for C_20_H_13_N_3_O_4_: C, 68.87; H, 3.76; N, 8.03. Found: C, 68.63; H, 3.40; N 8.23. 

*2-(4-(Benzyloxy)phenyl)-5-(4-phenoxyphenyl)-1,3,4-oxadiazole* (**6f**). White solid, Yield; 80%, m.p.: 226–229 °C. ^1^H-NMR (DMSO-*d*_6_) δ: 5.14 (s, 2H, OCH_2_-), 7.03 (d, *J* = 8.3 Hz, 2H, ArH), 7.06 (d, *J* = 8.1 Hz, 2H, ArH), 7.12 (t, *J* = 11.5 Hz, 2H, ArH), 7.34–7.45 (m, 8H, ArH), 7.99 (d, *J* = 8.6 Hz, 2H, ArH), 8.10 (d, *J* = 9.1 Hz, 2H, ArH). ^13^C-NMR (DMSO-*d*_6_) δ: 70.9 (OCH_2_-), 114.6 (C3, C5) 114.8 (C3′, C5′), 115.7 (C3″), 118.0 (C5″), 118.6 (C3‴, C5‴), 121.3 (C1, C1′), 127.4 (C1″, C1‴), 128.2 (C2, C6), 128.8 (C2′), 129.1 (C6′), 131.2 (C2″, C6″), 136.7 (C2‴, C6‴), 153.5 (C4, C4′), 155.2 (C4″), 157.3 (C4‴), 165.7 (C=N), 166.4 (C=N). Anal. Calcd for C_27_H_20_N_2_O_3_: C, 77.13; H, 4.79; N, 6.66. Found: C, 77.53; H, 4.60; N 6.24. 

*2-(Naphthalen-1-yl)-5-(4-phenoxyphenyl)-1,3,4-oxadiazole* (**6g**). White solid, Yield; 70%, m.p.: 173–175 °C. ^1^H-NMR (CDCl_3_) δ: 7.11–7.15 (m, 2H, ArH), 7.20–7.23 (m, 1H, ArH), 7.40–7.44 (m, 1H, ArH), 7.59–7.64 (m, 3H, ArH), 7.69–7.75, (m, 1H, ArH), 7.49–8.09 (m, 3H, ArH), 8.16 (d, *J* = 8.0 Hz, 1H, ArH), 8.26 (d, *J* = 7 Hz, 1H, ArH), 8.35 (d, *J* = 7 Hz, 1H, ArH), 9.28 (d, *J* = 8.5 Hz, 1H, ArH), 9.38 (d, *J* = 8.5 Hz, 1H, ArH). ^13^C-NMR (CDCl_3_) δ: 118.6, 120.2, 120.8, 124.8, 125.1, 126.5, 127.0, 128.45, 128.50, 128.8, 128.9, 129.2, 130.3, 132.7, 132.9, 134.1, 134.2, 155.9, 161.1 (Ar-C), 164.1 (C=N), 164.6 (C=N). Anal. Calcd for C_24_H_16_N_2_O_2_: C, 79.11; H, 4.43; N, 7.69. Found: C, 78.93; H, 4.40; N 8.01. 

*2-(4-Phenoxyphenyl)-5-(pyridin-3-yl)-1,3,4-oxadiazole* (**6h**). White solid, Yield; 70%, m.p.: 143–144 °C. ^1^H-NMR (DMSO-*d*_6_) δ: 7.03 (d, *J* = 7.5 Hz, 2H, ArH), 7.06 (d, *J* = 8.2 Hz, 2H, ArH), 7.19–7.22 (m, 3H, ArH), 7.39 (t, *J* = 9.0 Hz, 2H, ArH), 7.98 (d, *J* = 7.5 Hz, 2H, ArH), 8.83 (d, *J* = 8.0 Hz, 2H, ArH); ^13^C-NMR (DMSO-*d*_6_) δ: 114.9 (C3, C5), 115.1 (C3′, C5′), 118.3 (C5″-pyridyl), 120.5 (C1, C1′), 121.6 (C1″-pyridyl), 123.5 (C2, C6), 127.3 (C2′, C6′), 129.4 (C6″-pyridyl), 131.2 (C4), 132.45 (C4′-pyridyl), 151.07 (C2=N pyridyl, C4=N-pyridyl), 162.92 (C=N), 165.62 (C=N). Anal. Calcd for C_19_H_13_N_3_O_2_: C, 72.37; H, 4.16; N, 13.33. Found: C, 72.66; H, 4.39; N 13.65. 

*2-(4-Phenoxyphenyl)-5-(pyridin-4-yl)-1,3,4-oxadiazole* (**6i**). White solid, Yield; 70%, m.p.: 125–127 °C. ^1^H-NMR (CDCl_3_) δ: 7.05–7.16 (m, 6H, ArH), 7.22–7.26 (m, 1H, ArH), 7.41–7.45 (m, 3H, ArH), 8.08–8.15 (m, 3H, ArH). ^13^C-NMR (CDCl_3_) δ: 117.5 (C3), 117.6 (C5), 117.9 (C3′), 118.2 (C5′), 118.5 (C2, C6-pyridyl), 120.4 (C1), 120.5 (C1′), 120.7 (C1″-pyridyl), 124.9 (C2), 129.5 (C6), 130.3 (C2′), 130.4 (C6′), 131.1 (C4), 132.6 (C4′), 133.1 (C3=N pyridyl), 134.4 (C5=N pyridyl), 162.7 (2C=N). Anal. Calcd for C_19_H_13_N_3_O_2_: C, 72.37; H, 4.16; N, 13.33. Found: C, 72.54; H, 4.40; N 13.43. 

*2-(Furan-2-yl)-5-(4-phenoxyphenyl)-1,3,4-oxadiazole* (**6j**). White solid, Yield; 77%, m.p.: 129–132 °C. ^1^H-NMR (CDCl_3_) δ: 6.65 (s, 1H, ArH), 7.11–7.13 (m, 4H, ArH), 7.23–7.25 (m, 2H, ArH), 7.44 (t, *J* = 8.01, 2H, ArH), 7.70 (s, 1H, ArH), 8.10 (d, *J* = 8.0 Hz, 2H, ArH). ^13^C-NMR (CDCl_3_) δ: 112.4 (C3), 114.2 (C5), 118.1 (C3′), 118.5 (C5′), 120.2 (C1), 120.3 (C1′), 124.8 (C2-furfuryl), 129.0 (C5-furfuryl), 129.2 (C3, C4 furfuryl), 130.3 (C2, C6), 139.8 (C2′), 145.9 (C6′), 155.8 (C4), 157.5 (C4′), 161.2 (C=N), 163.9 (C=N). Anal. Calcd for C_18_H_12_N_2_O_3_: C, 71.05; H, 4.67; N, 9.21. Found: C, 71.36; H, 4.39; N 9.55. 

*2-(4-Phenoxyphenyl)-5-(thiophen-2-yl)-1,3,4-oxadiazole* (**6k**). White solid, Yield; 75%, m.p.: 139–141 °C. ^1^H-NMR (CDCl_3_) δ: 6.80–6.93 (m, 4H, ArH), 7.09–7.11 (m, 2H, ArH), 7.19–7.22 (m, 1H, ArH), 7.47–7.35 (m, 1H, ArH), 8.07–8.09 (m, 1H, ArH), 7.81–7.83 (m, 1H, ArH). ^13^C-NMR (CDCl_3_) δ: 118.2 (C3), 118.4 (C5), 120.2 (C3′), 124.7 (C5′), 125.4 (C1), 128.3 (C1′), 128.9 (C2, C5-thienyl), 129.0 (C3, C4-thienyl), 129.8 (C2), 130.0 (C6), 130.19 (C2′), 130.21 (C6′), 130.4 (C4), 155.8 (C4′), 161.0 (C=N), 163.9 (C=N). Anal. Calcd for C_18_H_12_N_2_O_2_S: C, 67.48; H, 3.78; N, 8.74. Found: C, 67.35; H, 3.36; N 9.02. 

*2,5-bis(4-Phenoxyphenyl)-1,3,4-oxadiazole* (**6l**). White solid, Yield; 85%, m.p.: 232–234 °C. ^1^H-NMR (CDCl_3_) δ: 6.95–7.12 (m, 9H, ArH), 7.17–7.24 (m, 2H, ArH), 7.32–7.40 (m, 4H, ArH), 8.04–8.406 (m, 3H, ArH). ^13^C-NMR (CDCl_3_) δ: 118.1, 118.5, 118.6, 118.9, 119.3, 119.5, 120.2, 123.9, 124.7, 129.0, 129.5, 129.8, 130.0, 130.3, 155.9 (Ar-C) 160.9 (C=N), 164.2 (C=N). Anal. Calcd for C_26_H_18_N_2_O_3_: C, 76.83; H, 4.46; N, 6.89. Found: C, 77.01; H, 4.60; N 6.74. 

*(E)-N′-Benzylidene-4-phenoxybenzohydrazide* (**8a**). White solid, Yield; 89%, m.p.: 163–165 °C. ^1^H-NMR (DMSO-*d*_6_) δ: 7.08–7.12 (m, 4H, ArH), 7.12–7.24 (m, 1H, ArH), 7.44–7.47 (m, 5H, ArH), 7.73 (d, *J* = 6.9 2H, ArH), 7.96 (d, *J* = 8.2 Hz, 2H, ArH), 8.46 (s, 1H, CH=N), 11.82 (s, 1H, NH). ^13^C-NMR (DMSO-*d*_6_) δ: 117.5 (C3, C5), 119.6 (C3′, C5′), 124.4 (C3″, C5″), 127.1 (C1, C1′), 127.9 (C1″), 128.8 (C2, C6), 128.9 (C2′, C6′), 130.0 (C2″), 130.3 (C6″), 134.4 (C4), 147.5 (C4′), 155.5 (C4″), 159.9 (C=O), 162.4 (C=N). Anal. Calcd for C_20_H_16_N_2_O_2_: C, 75.93; H, 5.10; N, 8.86. Found: C, 75.54; H, 5.32; N 9.11. 

*(E)-4-Phenoxy-N′-(3,4,5-trimethoxybenzylidene)benzohydrazide* (**8b**). White solid, Yield; 92%, m.p.: 191–192 °C. ^1^H-NMR (DMSO-*d*_6_) δ: 3.71 (s, 3H, OMe), 3.84 (s, 6H, 2OMe), 7.03 (s, 2H, ArH), 7.10 (d, *J* = 7.9 Hz, 4H, ArH), 7.21–7.24 (m, 1H, ArH), 7.45 (t, *J* = 7.9 Hz, 2H, ArH), 7.95 (d, *J* = 8.0 Hz, 2H, ArH), 8.39 (s, 1H, CH=N), 11.80 (s, 1H, NH). ^13^C-NMR (DMSO-*d*_6_) δ: 55.9 (OMe), 602.1 (2OMe), 104.3 (C3, C5), 117.5 (C3′, C5′), 119.6 (C3″, C5″), 124.4 (C1, C1′), 128.0 (C1″), 129.9 (C2, C6), 130.3 (C2′, C6′), 139.2 (C2″, C6″), 147.6 (C4), 153.2 (C4′), 155.5 (C4″), 159.9 (C=O), 162.4 (C=N). Anal. Calcd for C_23_H_22_N_2_O_5_: C, 67.97; H, 5.46; N, 6.89. Found: C, 67.92; H, 5.32; N 7.02.

*(E)-N′-(4-Methoxybenzylidene)-4-phenoxybenzohydrazide* (**8c**). White solid, Yield; 92%, m.p.: 233–235 °C. ^1^H-NMR (DMSO-*d*_6_) δ: 3.81 (s, 3H, OMe), 7.02 (d, *J* = 8.4, 2H, ArH), 7.08–7.12 (m, 4H, ArH), 7.22 (t, *J* = 7.5 Hz, 1H, ArH), 7.45 (t, *J* = 8.0 Hz, 2H, ArH), 7.67 (d, *J* = 8.0 Hz, 2H, ArH), 7.95 (d, *J* = 8.2 Hz, 2H, ArH), 8.40 (s, 1H, CH=N), 11.68 (s, 1H, NH). ^13^C-NMR (DMSO-*d*_6_) δ: 55.3 (OMe), 114.3 (C3, C5), 117.5 (C3′, C5′), 119.6 (C3″, C5″), 124.4 (C1, C1′), 126.9 (C1″), 128.1 (C2, C6), 128.6 (C2′, C6′), 129.8 (C2″), 130.3 (C6″), 147.4 (C4), 155.5 (C4′), 159.8 (C4″), 160.8 (C=O), 162.2 (C=N). Anal. Calcd for C_21_H_18_N_2_O_3_: C, 72.82; H, 5.24; N, 8.09. Found: C, 72.72; H, 5.53; N 8.06. 

*(E)-N′-(4-Chlorobenzylidene)-4-phenoxybenzohydrazide* (**8d**). White solid, Yield; 92%, m.p.: 184–186 °C. ^1^H-NMR (DMSO-*d*_6_) δ: 7.06 (d, *J* = 7 Hz, 2H, ArH), 7.09 (d, *J* = 7.5 Hz, 2H, ArH), 7.22 (t, *J* = 11.5 Hz, 2H, ArH), 7.44 (t, *J* = 12.0 Hz, 3H, ArH), 7.99 (d, *J* = 8.4 Hz, 2H, ArH), 8.10 (d, *J* = 9.2 Hz, 2H, ArH), 8.77 (s, 1H, CH=N), 11.86 (s, 1H, NH). ^13^C-NMR (DMSO-*d*_6_) δ: 117.5 (C3, C5), 119.6 (C3′, C5′), 124.4 (C3″, C5″), 127.8 (C3‴, C5‴), 128.7 (C1, C1′), 128.9 (C1″, C1‴), 129.9 (C2, C6), 130.3 (C2′, C6′), 133.3 (C2″, C6″), 134.4 (C2‴, C6‴), 146.2 (C4, C4′), 155.4 (C4″, C4‴), 160.0 (C=O), 162.5 (C=N). Anal. Calcd for C_20_H_15_ClN_2_O_2_: C, 68.48; H, 4.31; N, 7.99. Found: C, 68.37; H, 4.40; N 8.33. 

*(E)-N′-(4-Chlorobenzylidene)-4-phenoxybenzohydrazide* (**8e**). White solid, Yield; 92%, m.p.: 184–186 °C. ^1^H-NMR (DMSO-*d*_6_) δ: 7.09–7.12 (m, 4H, ArH), 7.23 (t, *J* = 7.5 Hz, 2H, ArH), 7.45 (t, *J* = 7.5 Hz, 2H, ArH), 7.97 (d, *J* = 8.0 Hz, 4H, ArH), 8.29 (d, *J* = 8.4 Hz, 2H, ArH), 8.54 (s, 1H, CH=N), 12.10 (s, 1H, NH).^13^C-NMR (DMSO-*d*_6_) δ: 117.4 (C3, C5), 119.7 (C3′, C5′), 124.1 (C3″, C5″), 124.5 (C3‴, C5‴), 127.5 (C1, C1′), 127.9 (C1″, C1‴), 130.1 (C2, C6), 130.3 (C2′, C6′), 140.7 (C2″, C6″), 145.0 (C2‴, C6‴), 147.8 (C4, C4′), 148.8 (C4″), 155.4 (C4‴), 160.2 (C=O), 162.6 (C=N). Anal. Calcd for C_20_H_15_ClN_2_O_2_: C, 68.48; H, 4.31; N, 7.99. Found: C, 68.37; H, 4.40; N 8.33. 

*(E)-N′-[4-(Benzyloxy)benzylidene]-4-phenoxybenzohydrazide* (**8f**). White solid, Yield; 93%, m.p.: 228–231 °C. ^1^H-NMR (DMSO-*d*_6_) δ: 5.17 (s, 2H, ArH), 7.08–7.12 (m, 6H, ArH), 7.21–7.24 (m, 1H, ArH), 7.33–7.48 (m, 7H, ArH), 7.67 (d, *J* = 8.7 Hz, 2H, ArH), 7.94 (d, *J* = 8.6 Hz, 2H, ArH), 8.39 (s, 1H, CH=N), 11.68 (s, 1H, NH). ^13^C-NMR (DMSO-*d*_6_) δ: 69. 4 (OCH_2_), 115.2 (C3), 117.5 (C5), 119.6 (C3′, C5′), 124.4 (C3″), 127.1 (C5″), 127.8 (C1), 127.9 (C1′), 128.1 (C1″), 128.5 (C2), 128.6 (C6), 129.8 (C2′, C6′), 130.3 (C2″, C6″), 136.8 (C4), 147.4 (C4′), 155.5 (C4″), 159.9 (C=O), 162.2 (C=N). Anal. Calcd for C_27_H_22_N_2_O_3_: C, 76.76; H, 5.25; N, 6.63. Found: C, 76.56; H, 5.60; N 6.34. 

*(E)-N′-(Naphthalen-1-ylmethylene)-4-phenoxybenzohydrazide* (**8g**). White solid, Yield; 88%, m.p.: 193–195 °C. ^1^H-NMR (DMSO-*d*_6_) δ: 7.12–7.14 (m, 4H, ArH), 7.24 (t, *J* = 7.9 Hz, 2H, ArH), 7.45–7.48 (m, 2H, ArH), 7.60–7.62, (m, 2H, ArH), 7.63–7.69, (m, 1H, ArH), 7.93–7.95 (m, 1H, ArH), 8.01–8.04 (m, 4H, ArH), 8.87–8.89 (m, 1H, ArH), 9.11 (s, 1H, CH=N), 11.90 (s, 1H, NH). ^13^C-NMR (DMSO-*d*_6_) δ: 117.5, 119.6, 124.2, 124.4, 125.6, 126.3, 127.3, 127.7, 127.9, 128.8, 129.6, 129.9, 130.2, 130.3, 130.5, 133.5, 147.4, 155.5 (Ar-C), 160.0 (C=O), 162.3 (C=N). Anal. Calcd for C_24_H_18_N_2_O_2_: C, 78.67; H, 4.95; N, 7.65. Found: C, 78.92; H, 4.60; N 8.04. 

*(E)-4-Phenoxy-N′-(pyridin-3-ylmethylene)benzohydrazide* (**8h**). White solid, Yield; 90%, m.p.: 149–151 °C. ^1^H-NMR (DMSO-*d*_6_) δ: 7.09–7.14 (m, 4H, ArH), 7.22–7.25 (m, 1H, ArH), 7.44–7.50 (m, 3H, ArH), 7.97 (d, *J* = 8.4 Hz, 2H, ArH), 8.14 (d, *J* = 7.0 Hz, 1H, ArH), 8.51 (s, 1H, CH=N), 8.62 (d, *J* = 8.3 Hz, 1H, ArH), 8.86 (d, *J* = 8.3 Hz, 1H, ArH), 11.98 (s, 1H, NH). ^13^C-NMR (DMSO-*d*_6_) δ: 117.5 (C3, C5), 119.6 (C3′, C5′), 124.0 (C5-pyridyl), 124.5 (C1), 127.7 (C1′), 130.0 (C1-pyridyl), 130.3 (C2, C6), 133.4 (C2′, C6′), 144.8 (C6-pyridyl), 148.7 (C4, C4′), 150.7 (C2=N pyridyl), 155.4 (C4=N pyridyl), 160.0 (C=O), 162.5 (C=N). Anal. Calcd for C_19_H_15_N_3_O_2_: C, 71.91; H, 4.76; N, 13.24. Found: C, 71.46; H, 4.54; N 13.55. 

*(E)-4-Phenoxy-N′-(pyridin-4-ylmethylene)benzohydrazide* (**8i**). White solid, Yield; 92%, m.p.: 175–177 °C. ^1^H-NMR (DMSO-*d*_6_) δ: 7.10–7.13 (m, 4H, ArH), 7.24 (t, *J* = 8.2 Hz, 1H, ArH), 7.46 (t, *J* = 8.2 Hz, 2H, ArH), 7.67 (s, 2H, ArH), 7.97 (d, *J* = 7.9 Hz, 2H, ArH), 8.44 (s, 1H, CH=N), 8.66 (s, 2H, ArH), 12.08 (s, 1H, NH). ^13^C-NMR (DMSO-*d*_6_) δ: 117.5 (C3, C5), 119.7 (C3′, C5′), 121.0 (C5-pyridyl), 124.5 (C1, C1′), 127.5 (C1-pyridyl), 130.1 (C2, C6), 130.3 (C2′, C6′), 141.5 (C6-pyridyl), 145.0 (C4, C4′), 150.3 (C3=N pyridyl), 155.4 (C4=N pyridyl), 160.2 (C=O), 162.6 (C=N). Anal. Calcd for C_19_H_15_N_3_O_2_: C, 71.91; H, 4.76; N, 13.24. Found: C, 71.64; H, 4.60; N 13.42. 

*(E)-N′-(Furan-2-ylmethylene)-4-phenoxybenzohydrazide* (**8j**). White solid, Yield; 89%, m.p.: 192–194 °C. ^1^H-NMR (DMSO-*d*_6_): δ 6.64 (s, 1H, ArH), 6.92 (s, 1H, ArH), 7.08–7.12 (m, 4H, ArH), 7.21–7.24 (m, 1H, ArH), 7.45 (t, *J* = 7.9 Hz, 2H, ArH), 7.85 (s, 1H, ArH), 7.93 (d, *J* = 7.9 Hz, 2H, ArH), 8.34 (s, 1H, CH=N), 11.75 (s, 1H, NH). ^13^C-NMR (DMSO-*d*_6_) δ: 112.2 (C3), 133.4 (C5), 117.5 (C3′, C5′), 119.6 (C3, C4–furfuryl), 124.4 (C1), 127.9 (C1′), 129.9 (C2, C6), 130.3 (C2′, C6′), 137.3 (C2—furfuryl), 145.1 (C5–furfuryl), 149.5 (C4), 155.5 (C4′), 159.9 (C=O), 162.3 (C=N). Anal. Calcd for C_18_H_14_N_2_O_3_: C, 70.58; H, 4.61; N, 9.15. Found: C, 70.37; H, 4.32; N 9.50. 

*(E)-4-Phenoxy-N′-(thiophen-2-ylmethylene)benzohydrazide* (**8k**). White solid, Yield; 92%, m.p.: 182–183 °C. ^1^H-NMR (DMSO-*d*_6_) δ: 7.08–7.24 (m, 6H, ArH), 7.44–7.47 (m, 3H, ArH), 7.67 (d, *J* = 7.5 Hz, 1H, ArH), 7.93 (d, *J* = 7.0 Hz, 2H, ArH), 8.66 (s, 1H, CH=N), 11.76 (s, 1H, NH). ^13^C-NMR (DMSO-*d*_6_) δ: 117.5 (C3, C5), 119.6 (C3′, C5′), 124.4 (C3, C4 thienyl), 127.8 (C1) 128.8 (C1′), 128.8 (C2, C6), 129.8 (C2′), 130.3 (C6′) 130.8 (C4), 139.2 (C4′), 142.7 (C2–thienyl), 155.5 (C4–thienyl), 159.9 (C=O), 162.2 (C=N). Anal. Calcd for C_18_H_14_N_2_O_2_S: C, 67.06; H, 4.38; N, 8.69. Found: C, 67.36; H, 3.39; N 9.04. 

### 3.2. Biology

#### 3.2.1. In Vitro Anti-Tubercular Activity

The MICs of Compounds **3**, **4**, **6a**–**l**, and **8a**–**k** for M. tuberculosis H37Rv were determined by the microplate Alamar blue assay (MABA) (Table 1) [31,32]. Triclosan and INH were used as reference standards. *M. tuberculosis* H37Rv (ATCC 27294; American Type Culture Collection, Rockville, MD, USA) was grown to late log phase in Middlebrook 7H9 broth. Twofold dilutions of tested drugs were prepared in 7H9 broth in a volume of 100 μL in 96-well, clear-bottom microplates at a final testing concentration of 0.2, 0.4, 0.8, 1.6, 3.12, 6.25, 12.5, 25, 50 and 100 μg/mL. 100 μL of M. tuberculosis H37Rv (containing 2 × 10^4^ CFU) was added, yielding a final testing volume of 200 μL. In each row, a drug-free (bacteria-only) control was added.

The plates were incubated at 37 °C; on the seventh day of incubation, 12.5 μL of 20% Tween 80 and 20 μL of Alamar blue were added to all wells. After incubation at 37 °C for 16 to 24 h, the fluorescence of the wells was read at an excitation of 530 nm and emission of 590 nm. The MIC was defined as the lowest concentration affecting a reduction in fluorescence of ≥90% relative to the mean of replicate bacteria-only controls. 

Among the tested compounds, the results displayed that most of them i.e., **3**, **4**, **6h**, **6i**, **6l**, **8g**, and **8k** showed promising anti-tubercular activity (MIC, 0.61–1.69 μg/mL) in comparison to triclosan (MIC, 10 μg/mL) and INH (MIC, 0.2 μg/mL). 

#### 3.2.2. In Vitro InhA Enzyme Inhibition

InhA enzyme assay was carried out utilizing a Cary100 Bio spectrophotometer (Agilent Technologies, Santa Clara, California, USA) at 25 °C, through monitoring the oxidation of NADH to NAD^+^ at 340 nm. Reactions were initiated via the addition of dodecenoyl-CoA (50 mm) substrate to mixtures containing InhA (5 nm), NADH (100 mm), and inhibitor (1–10,000 nm). The IC_50_ values were determined from a dose-response plot of enzyme fractional activity versus the concentration of inhibitor.

#### 3.2.3. Cytotoxic Activity 

The cytotoxicity test was carried out using Vero cell line (African green monkey kidney epithelial cells), obtained from American Type Culture Collection (ATCC). The cells were cultured according to ATCC recommendations as it was described earlier [41,42]. To evaluate cytotoxicity, 3 × 10^4^ Vero cells were seeded in the wells of 96-multiwell plates in 100 µL of the complete culture medium. After 24 h incubation at 37 °C when the cells were well attached and achieved 90% confluence, the culture medium was replaced with 100 µL of derivative solutions, which were tested at the concentration range of 0.1–200 µg/mL. The stock solutions of the synthesized derivatives were prepared in DMSO and the tested concentrations were prepared by nine serial 2-fold dilutions. Vero cells were exposed to the compounds and rifampicin for 24 h and then cell viability was assessed by MTT assay (Sigma-Aldrich Chemicals, St. Louis, MO, USA). The IC_50_ value, which is defined as the concentration of the derivative causing reduction in cell viability by 50%, was calculated using GraphPad Prism 5, version 5.03 software. Selectivity/safety index (SI) for tested agents was determined using the formula: SI = IC_50_/MIC.

#### 3.2.4. Docking Methodology

Discovery Studio 2.5 software (Accelrys Inc., San Diego, CA, USA) was used for docking analysis. Fully automated docking tool using “Dock ligands (CDOCKER)” protocol running on Intel (R) Core (TM) i32370 CPU @ 2.4 GHz, RAM Memory 2 GB under the Windows 7.0 system. The crystal structures of InhA reductase enzyme (PDB code: 3FNH) were downloaded from the protein data bank [36,37,38]. See the Supplementary Materials.

## 4. Conclusions

A set of diphenyl ether hydrazides and their cyclized oxadiazole analogs were designed and synthesized as triclosan analogs. The targeted compounds have been identified using spectroscopic data and elemental microanalysis. The antitubercular activity of the un-substituted oxadiazole **3** and the hydrazide **4** experienced the highest activity than triclosan as a reference drug. Arylated oxadiazoles **6i** and **6l** and arylidenes **8g** and **8k** showed potent antitubercular activity against *Mycobacterium tuberculosis* H37Rv strains. The InhA reductase enzymatic study supports the experimental data. We believe there is more than one mode of mechanisms are involved in the antimycobacterial property of the synthesized diphenyl ether derivatives. The obtained results from the in silico pharmacokinetic properties calculation showed that most of the target compounds exhibited drug-like characters. Taken together, a docking study has boosted that the higher activity of compounds **3**, **4**, and **8g** could be further investigated as potential lead antimycobacterial candidates. 

## Figures and Tables

**Table 1:** The antitubercular activity of the diphenyl ether derivatives against H37Rv strain of *M. tuberculosis* (MIC (μg/mL) and their inhibition for InhA reductase enzyme (IC_50_ µM).

Ar	No.	MIC (μg/mL)	IC_50_ (µM)	No.	MIC (μg/mL)	IC_50_ (µM)
----------	**3**	0.86	3.43	**4**	0.61	3.28
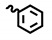	**6a**	2.74	>100	**8a**	34.54	73.23
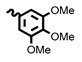	**6b**	34.64	>100	**8b**	50.20	>100
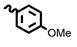	**6c**	39.04	>100	**8c**	20.68	75.46
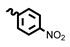	**6d**	15.15	68.44	**8d**	7.67	19.75
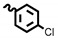	**6e**	15.89	64.55	**8e**	2.89	7.46
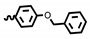	**6f**	8.23	41.24	**8f**	22.62	29.89
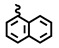	**6g**	12.23	33.82	**8g**	0.98	4.23
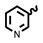	**6h**	1.40	5.08	**8h**	6.96	26.54
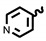	**6i**	1.40	11.72	**8i**	6.82	21.39
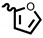	**6j**	11.12	51.42	**8j**	6.35	18.37
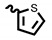	**6k**	3.65	78.65	**8k**	1.69	5.61
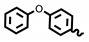	**6l**	1.17	5.63	**INH**	0.20	ND
----------	--------	----------	----------	**Triclosan**	10.00	1.10

**Table 2:** Correlation between calculated physicochemical parameters and biological activity.

Comp. No.	MIC (μg/mL)	Molecular Mass	Balaban Index	Molecular Topology: Wiener Index	Topological Diameter Bond (s)	Molar Refractivity, cm^3^/mol	Clog *p*	Heat of Formation [kJ/mol]	Gibbs Energy [kJ/mol] Energy	H Bond Donor	H Bond Acceptor	Lipinski’s Rule
**3**	0.86	228.250	118,297	586	11	6.589	2.231	−60.70	176.14	2	3	Yes
**4**	0.61	238.250	121,561	682	11	6.656	2.753	115.57	339.72	0	4	Yes
**6a**	2.74	314.340	400,250	1561	15	9.167	4.806	216.79	493.02	0	4	Yes
**6b**	34.64	404.422	110,779	2828	17	11.018	4.427	−276.20	174.39	0	7	Yes
**6c**	39.04	344.370	59,322	1990	17	9.784	4.679	52.46	386.81	0	5	Yes
**6d**	15.15	359.340	71,066	2219	17	9.778	ND	ND	ND	0	5	Yes
**6e**	15.89	348.770	488,162	1763	16	9.659	5.364	189.58	471.46	0	4	No
**6f**	8.23	420.470	142,971	3769	21	12.295	6.412	165.15	549.74	0	5	No
**6g**	12.23	364.404	706,482	2391	17	10.855	5.803	313.83	623.72	0	4	No
**6h**	1.40	315.332	400,250	1561	15	8.956	3.468	270.22	561.65	0	5	No
**6i**	1.40	315.332	400,250	1561	15	8.956	3.468	270.22	561.65	0	5	Yes
**6j**	11.12	304.305	326,596	1380	14	8.381	3.421	74.450	372.20	0	5	Yes
**6k**	3.65	320.062	32,659	1380	14	8.976	4.788	251.71	498.18	0	4	Yes
**6l**	1.17	406.440	120,686	3377	20	11.831	6.343	185.97	541.32	0	5	No
**8a**	34.54	384.390	104,520	2849	19	10.994	4.734	145.52	ND	1	6	Yes
**8b**	50.20	474.470	250,732	4753	21	12.845	4.354	−347.47	ND	1	9	Yes
**8c**	20.68	414.420	145,706	3497	21	11.611	4.607	−18.810	ND	1	7	Yes
**8d**	7.67	414.420	145,706	3497	21	11.610	ND	−18.81	ND	1	7	Yes
**8e**	2.89	418.840	12,360	3158	20	11.485	5.292	118.31	ND	1	6	Yes
**8f**	22.62	490.519	301,096	6023	25	14.122	6.339	93.88	ND	1	7	No
**8g**	1.40	434.450	161,770	4015	20	12.682	5.730	242.56	ND	1	6	No
**8h**	6.96	385.380	1,045,201	2849	19	10783	3.396	198.95	ND	1	7	No
**8i**	6.82	385.383	1,045,201	2849	19	10.783	3.396	198.95	ND	1	7	Yes
**8j**	6.35	374.356	880,346	2566	18	10.208	3.349	3.180	ND	1	7	Yes
**8k**	1.69	390.417	88,034	2566	18	10.803	4.715	180.44	ND	1	6	Yes
**R**	-	0.308	−0.179	0.315	0.260	−0.100	0.152	−0.683	−0.602	−0.370	−0.360	-

**Table 3:** MIC, IC_50,_ and SI values determined for the selected most promising compounds.

Compound	*M. tuberculosis*	Vero Cell Line	SI (IC_50_/MIC)
	MIC, µg/mL	IC_50_ µg/mL ± SEM	
**3**	0.86	80.7 ± 1.31	93.83
**4**	0.61	99.4 ± 1.79	162.95
**6h**	1.40	91.4 ± 1.55	65.29
**6i**	1.40	95.5 ± 1.36	68.21
**6l**	1.17	92.6 ± 1.80	79.15
**8g**	0.98	72.2 ± 1.87	73.67
**6k**	1.69	68.6 ± 1.19	40.59

## Supplementary Materials

The following are available online: general experimental data and NMR spectra.
