# Supplementary material for: In Vitro Antimycobacterial Activity and Physicochemical Characterization of Diaryl Ether Triclosan Analogues as Potential InhA Reductase Inhibitors
Source: Molecules. 2020 Jul 8;25(14):3125. doi: 10.3390/molecules25143125 (PMC7397076; doi:10.3390/molecules25143125)

SUPPORTING INFORMATION

In vitro Antimycobacterial Activity and Physicochemical Characterization of Diaryl Ether Triclosan Analogues as Potential InhA Reductase Inhibitors

Tarek S. Ibrahim[1,2,*], Ehab S. Taher[3], Ebtihal Samir[4], Azizah M. Malebari[1], Ahdab N. Khayyat[1], Mamdouh F. A. Mohamed[5], Riham M. Bokhtia[2,6], Mohammed A. AlAwadh[1], Israa A. Seliem[2,6], Hani Z. Asfour[7], Nabil A. Alhakamy[8], Siva S. Panda[6,*] and Amany M.M. AL-Mahmoudy[2]

[1] Department of Pharmaceutical Chemistry, Faculty of Pharmacy, King Abdulaziz University, Jeddah, 21589, Saudi Arabia

[2] Department of Pharmaceutical Organic Chemistry, Faculty of Pharmacy, Zagazig University, Zagazig, 44519, Egypt

[3] Department of Pharmaceutical Organic Chemistry, Faculty of Pharmacy, Al-Azhar University, 71524, Assiut, Egypt

[4] Physical Chemistry, Department of Analytical Chemistry, Faculty of Pharmacy, Deraya University, New Minia 61519-Minia, Egypt

[5] Department of Pharmaceutical Chemistry, Faculty of Pharmacy, Sohag University, 82524 Sohag, Egypt

[6] Department of Chemistry & Physics, Augusta University, Augusta, GA 30912, USA

[7] Department of Medical Microbiology and Parasitology, Faculty of Medicine, King Abdulaziz University, Jeddah 21589, Kingdom of Saudi Arabia

[8] Department of Pharmaceutics, Faculty of Pharmacy, King Abdulaziz University, Jeddah 21589, Saudi Arabia

CONTENTS

Docking methodology **2**

Selected ^{1}H and ^{13}C NMR Spectra for some of our designed Compounds **3**

Docking methodology

Discovery Studio 2.5 software (Accelrys Inc., San Diego, CA, USA) was used for docking analysis. Fully automated docking tool using "Dock ligands (CDOCKER)" protocol running on Intel (R) core (TM) i32370 CPU @ 2.4 GHz 2.4 GHz, RAM Memory 2 GB under the Windows 7.0 system. The crystal structure of InhA reductase enzyme (PDB code: 3FNH) was downloaded from protein data bank [32-34]. The docked compounds were built using Chem. 3D ultra 12.0 software [Chemical Structure Drawing Standard; Cambridge Soft corporation, USA (2010)], and copied to Discovery Studio 2.5 software. Automatic protein preparation module was used applying CHARMm force field. The binding site sphere has been defined automatically by the software. Herein, the prepared receptor is given as input for "input receptor molecule" parameter in the CDOCKER protocol parameter explorer. Force fields are applied on compounds Triclosan**, 3**, **8b** and **8g** to get the minimum lowest energy structure. The obtained poses those have displayed the best ligand–InhA reductase enzyme interactions were selected and used for CDOCKER energy (protein-ligand interaction energies) calculations. Receptor–ligand interactions of the complexes were examined in 2D and 3D styles.

^{1}H NMR for compound 2

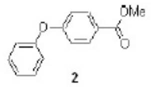

2

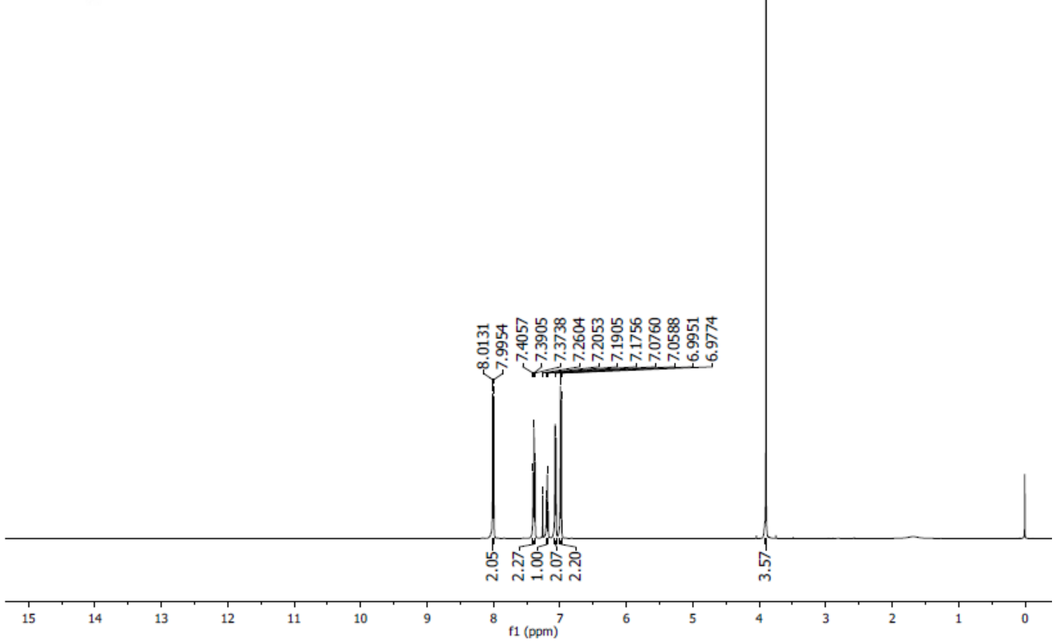

^{13}C NMR for compound 2

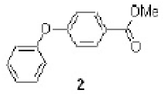

2

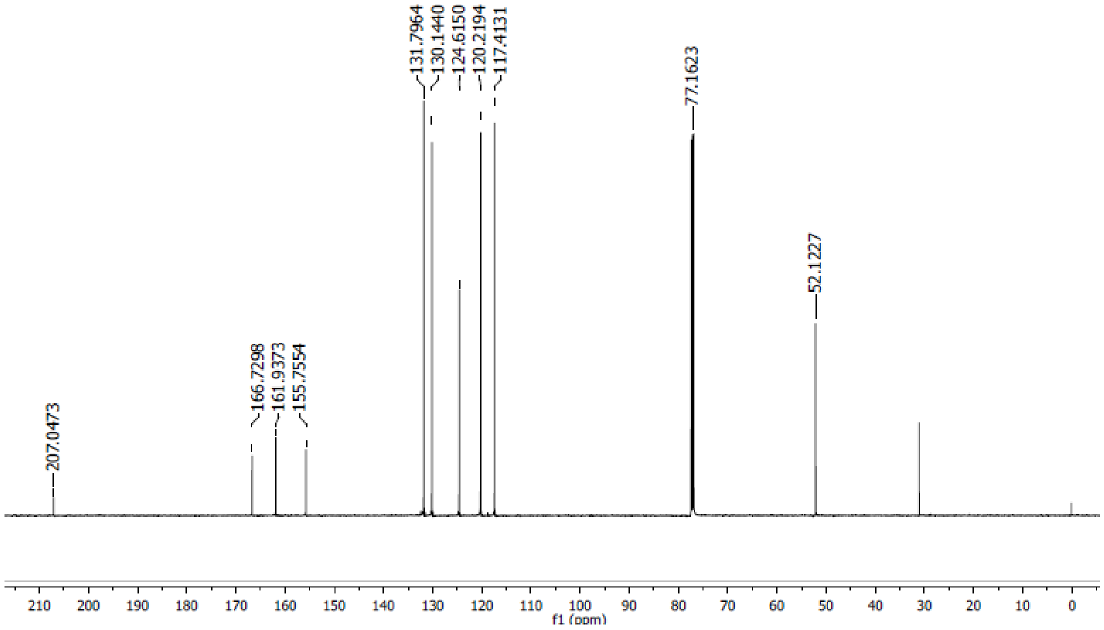

^{1}H NMR for compound 3

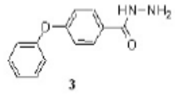

3

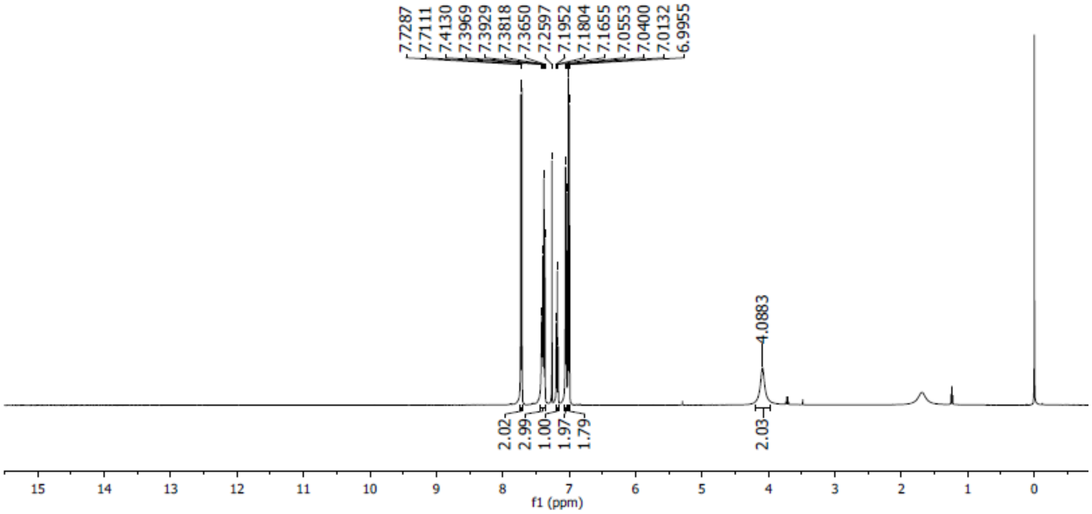

^{13}C NMR for compound 3

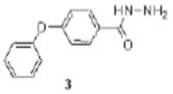

3

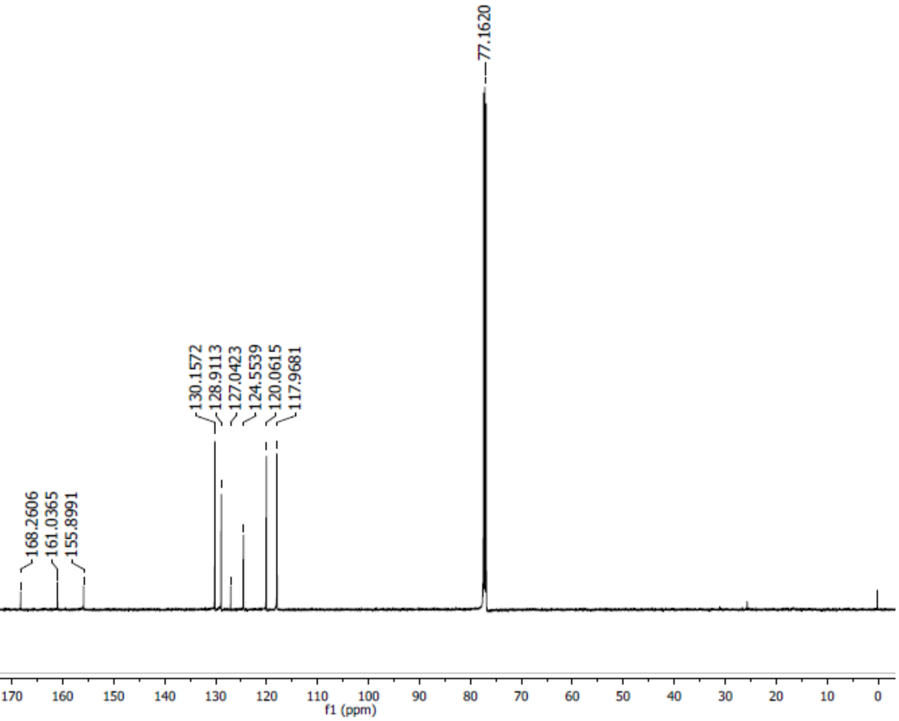

^{1}H NMR for compound 4

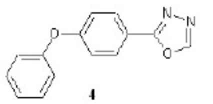

4

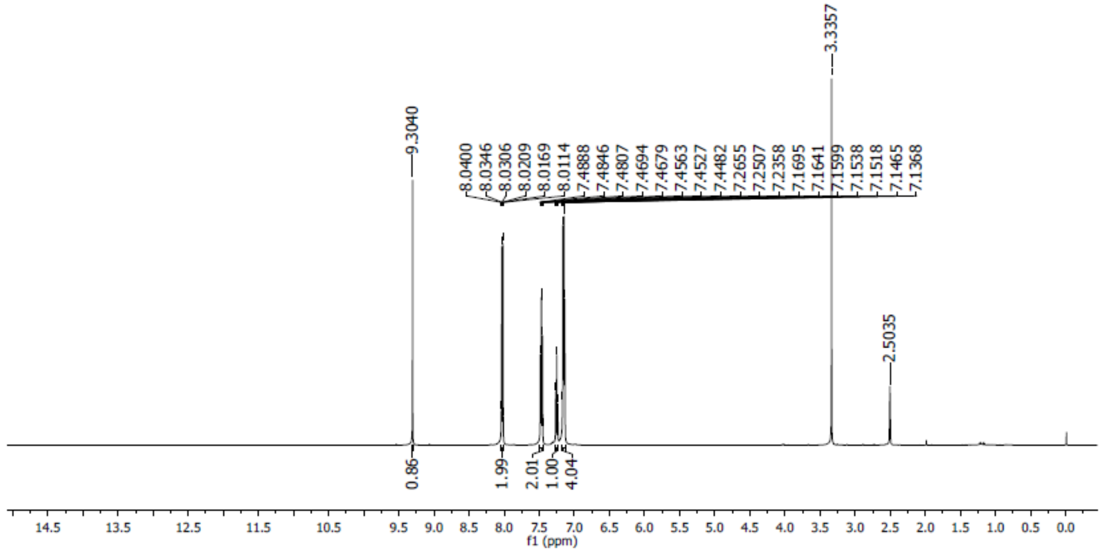

^{13}C NMR for compound 4

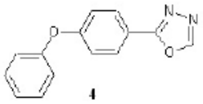

4

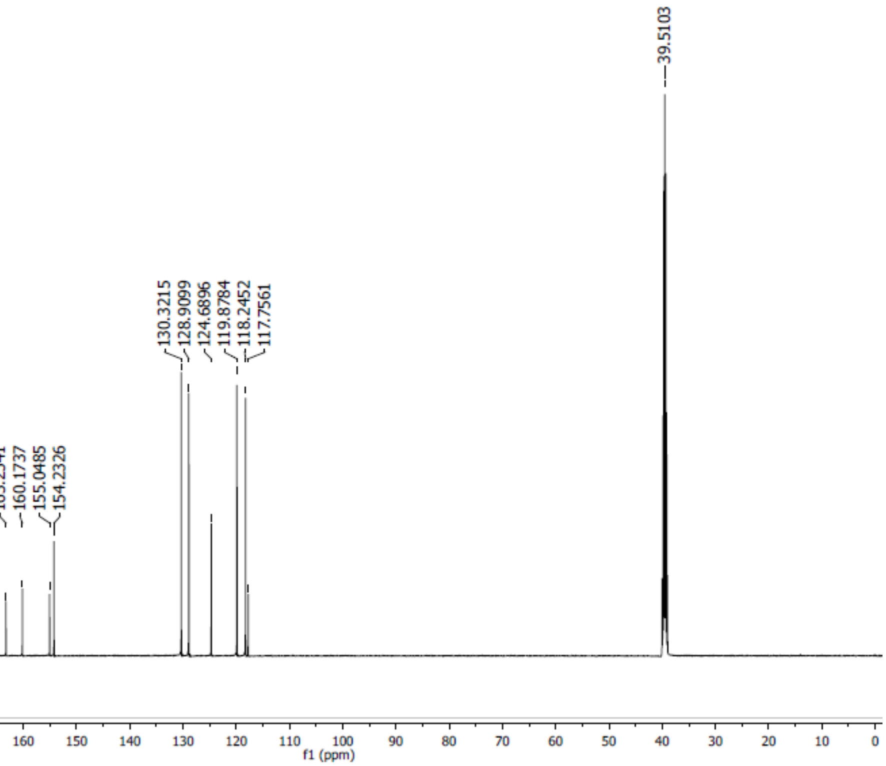

^{1}H NMR for compound 6a

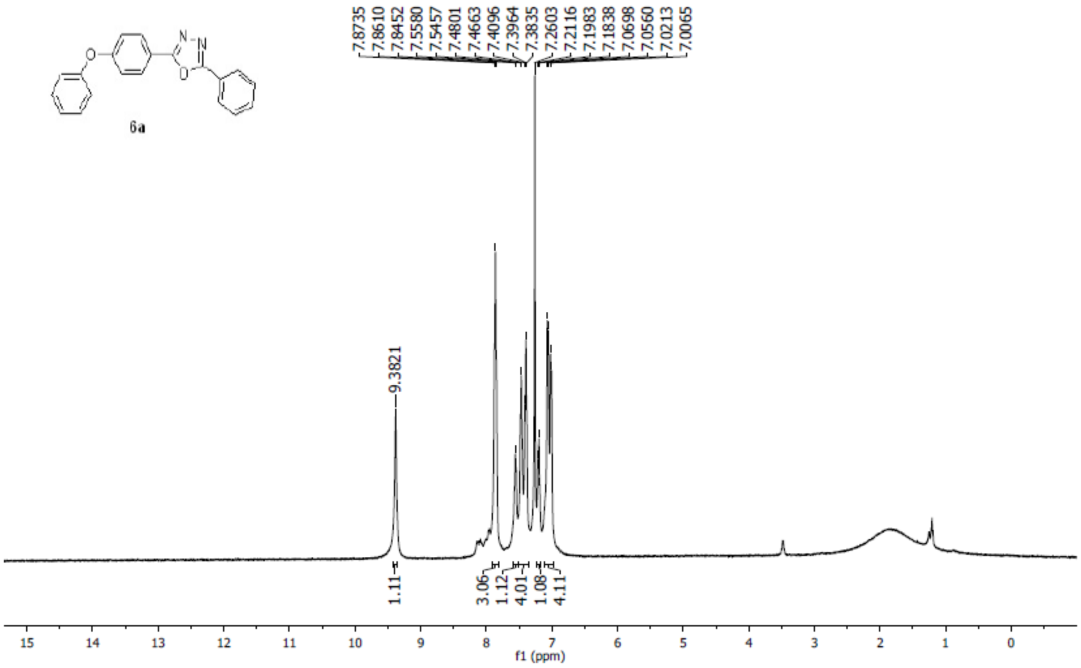

^{13}C NMR for compound 6a

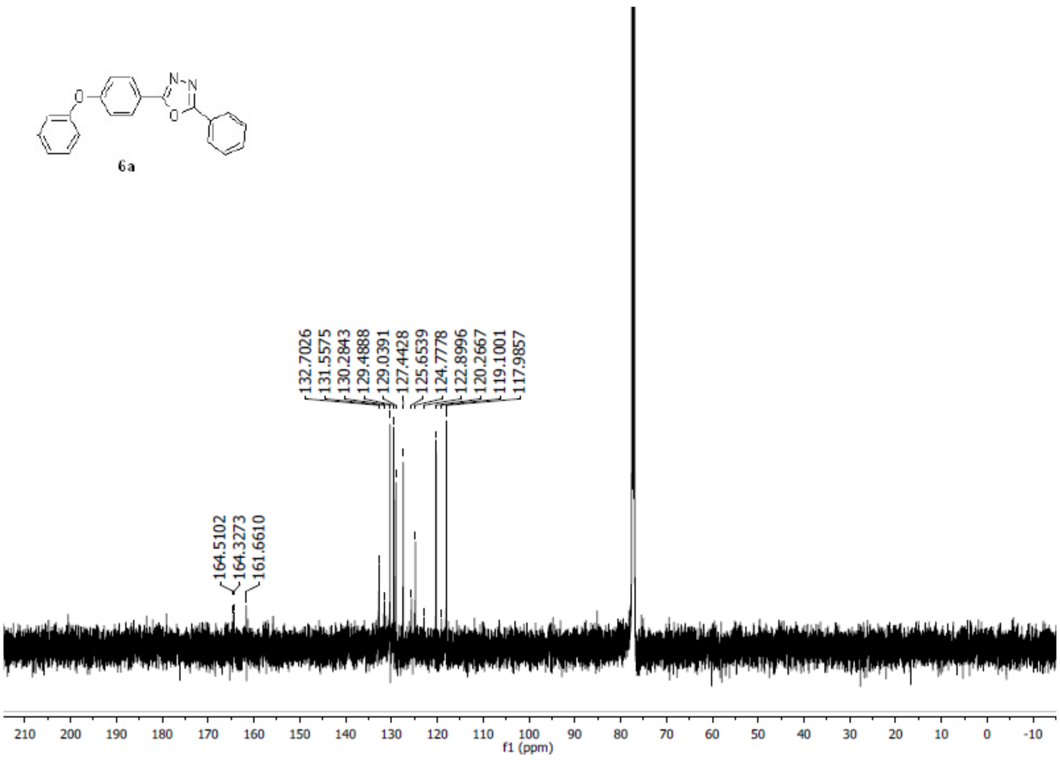

^{1}H NMR for compound 6b

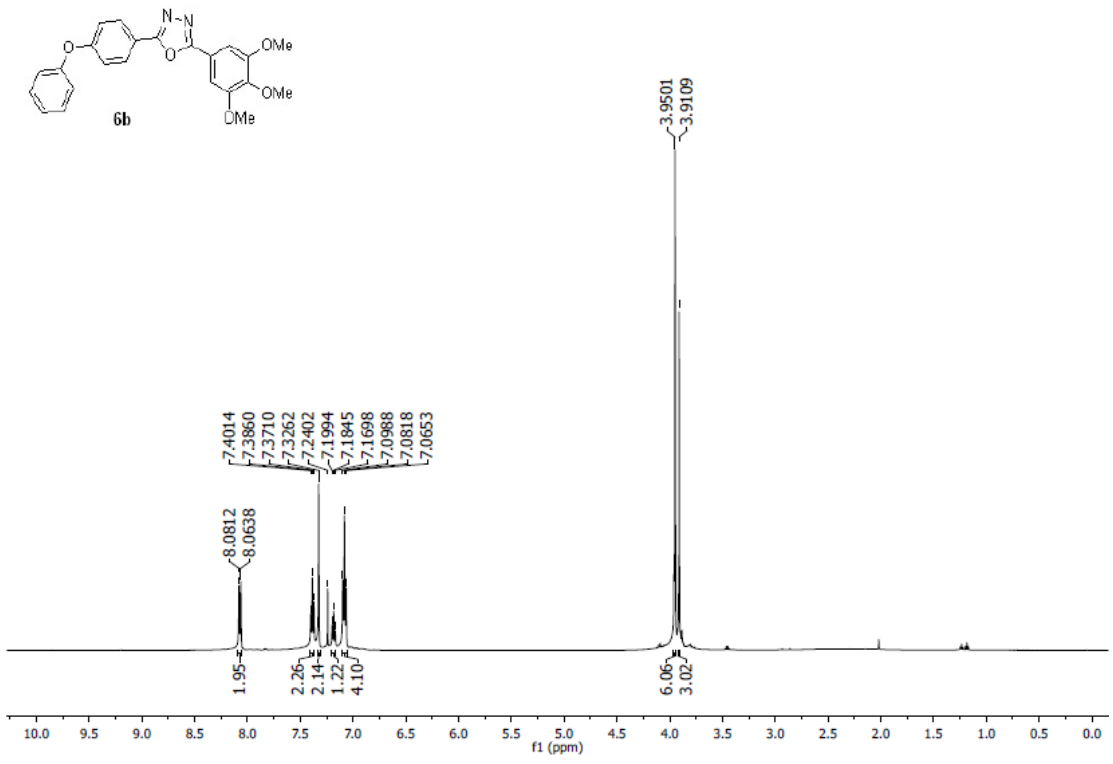

^{13}C NMR for compound 6b

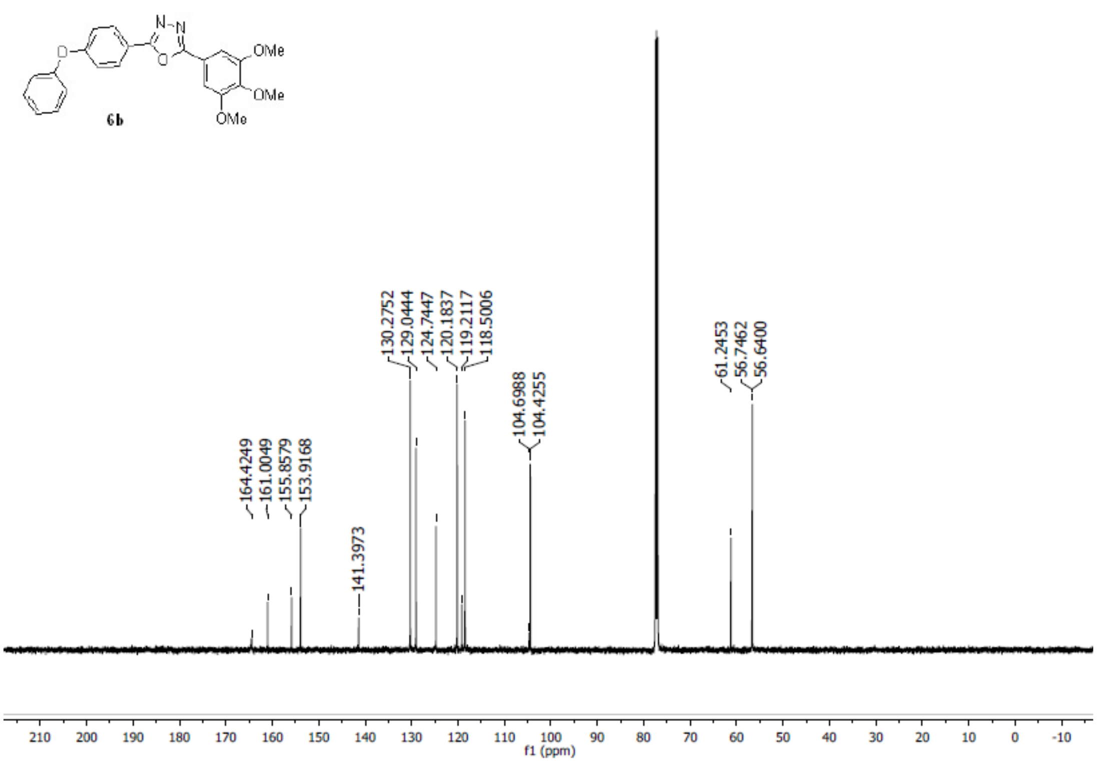

^{1}H NMR for compound 6c

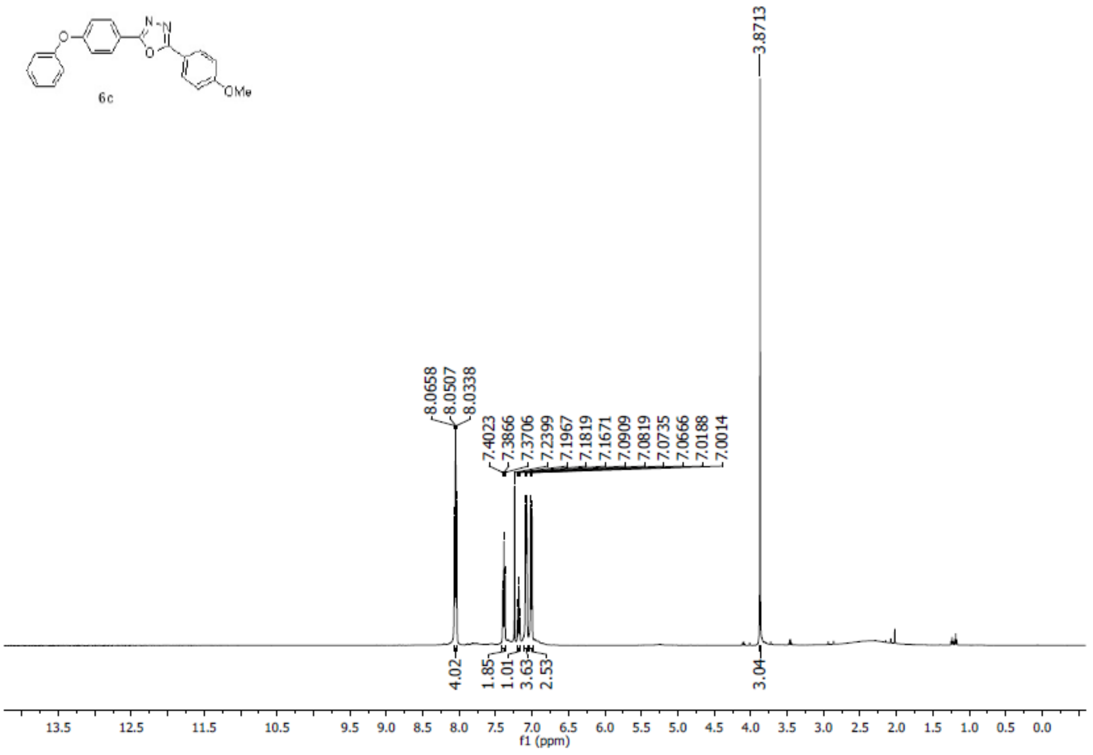

^{13}C NMR for compound 6c

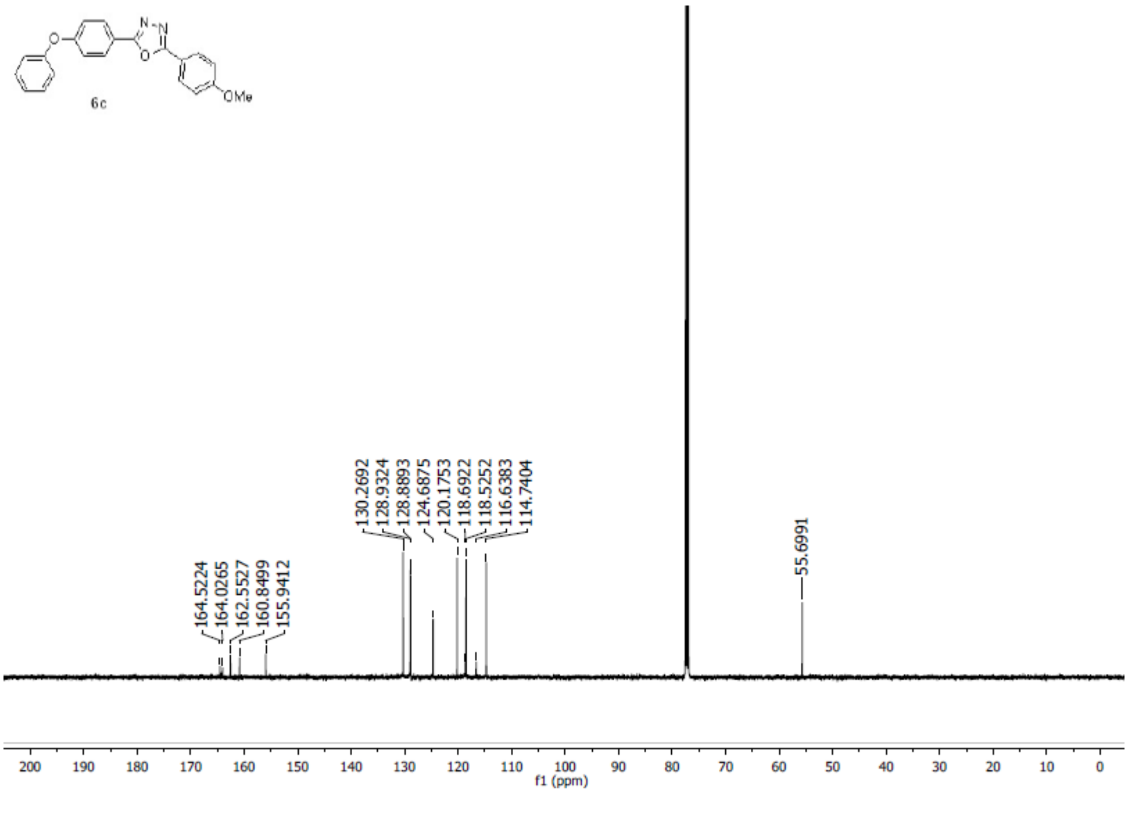

^{1}H NMR for compound 6d

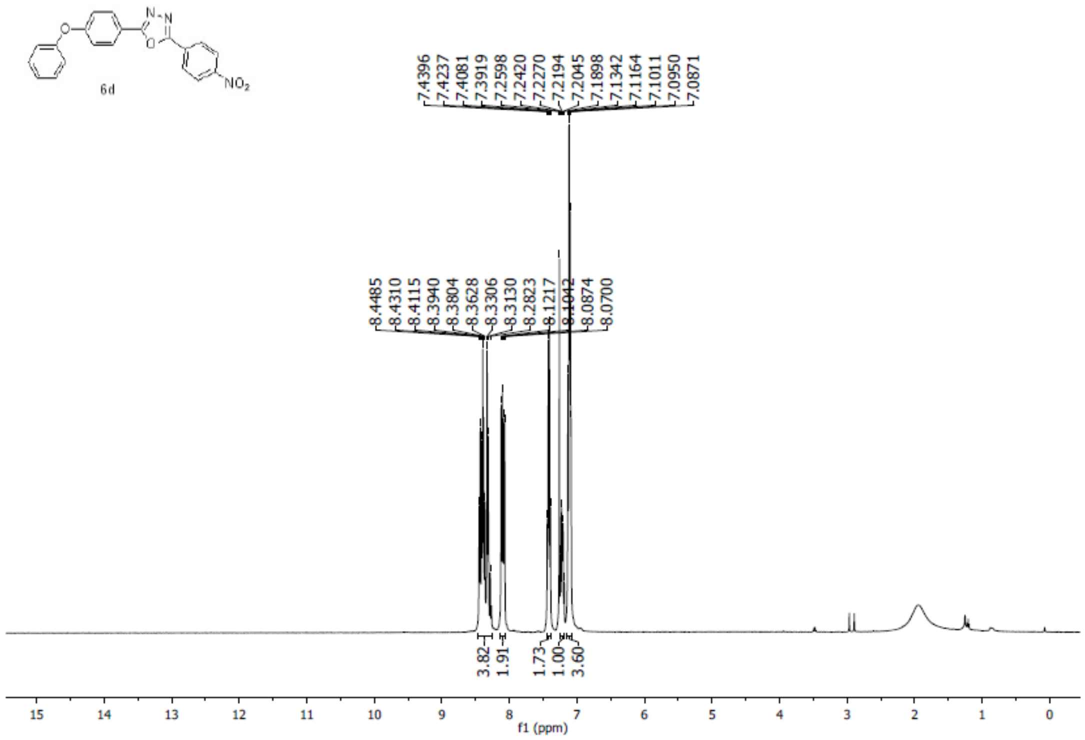

^{13}C NMR for compound 6d

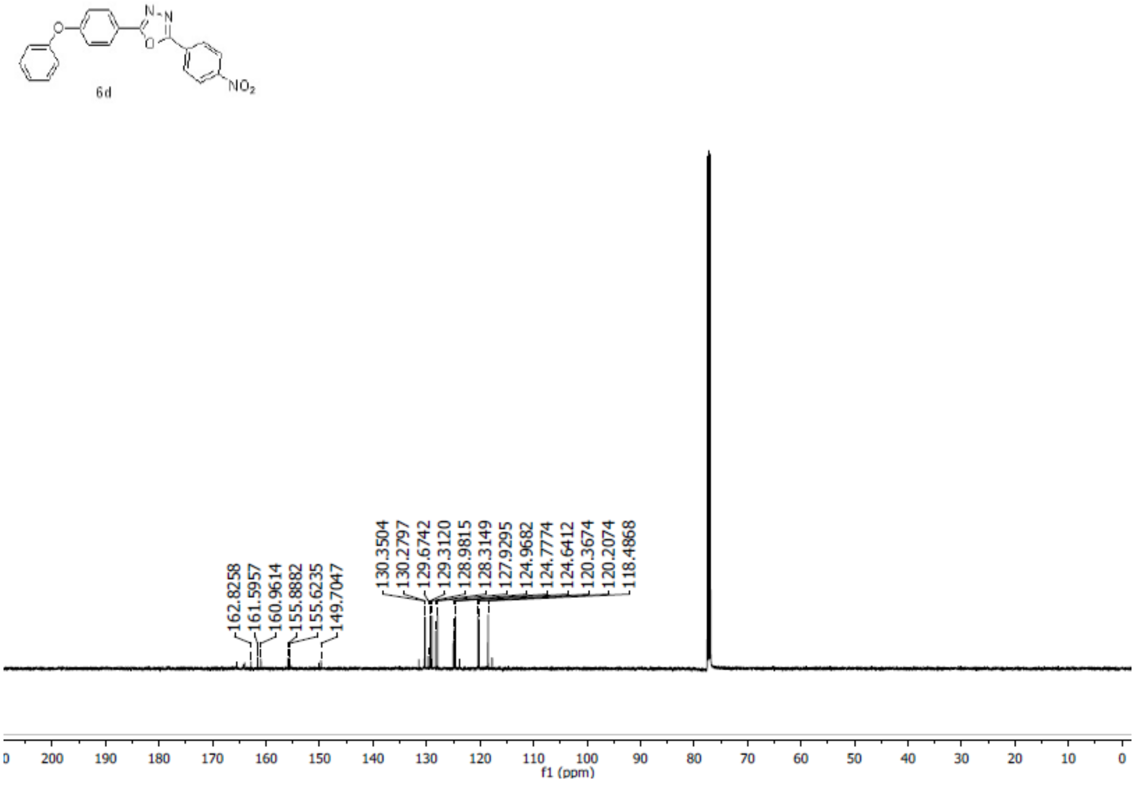

^{1}H NMR for compound 6e

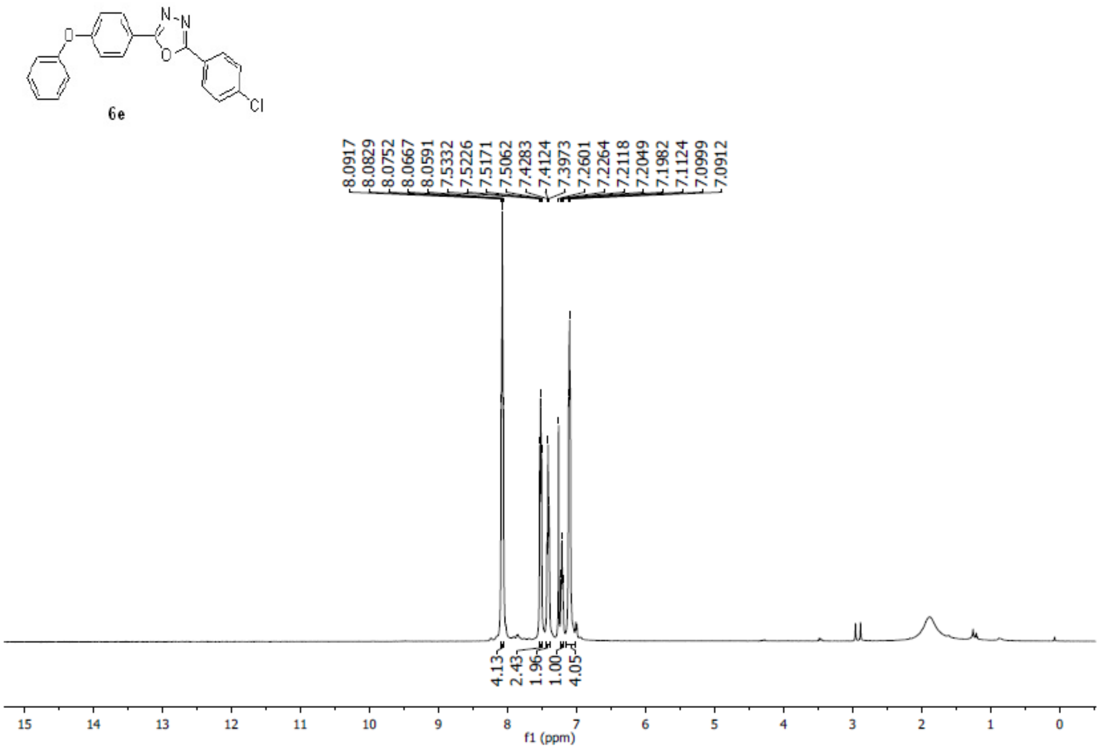

^{13}C NMR for compound 6e

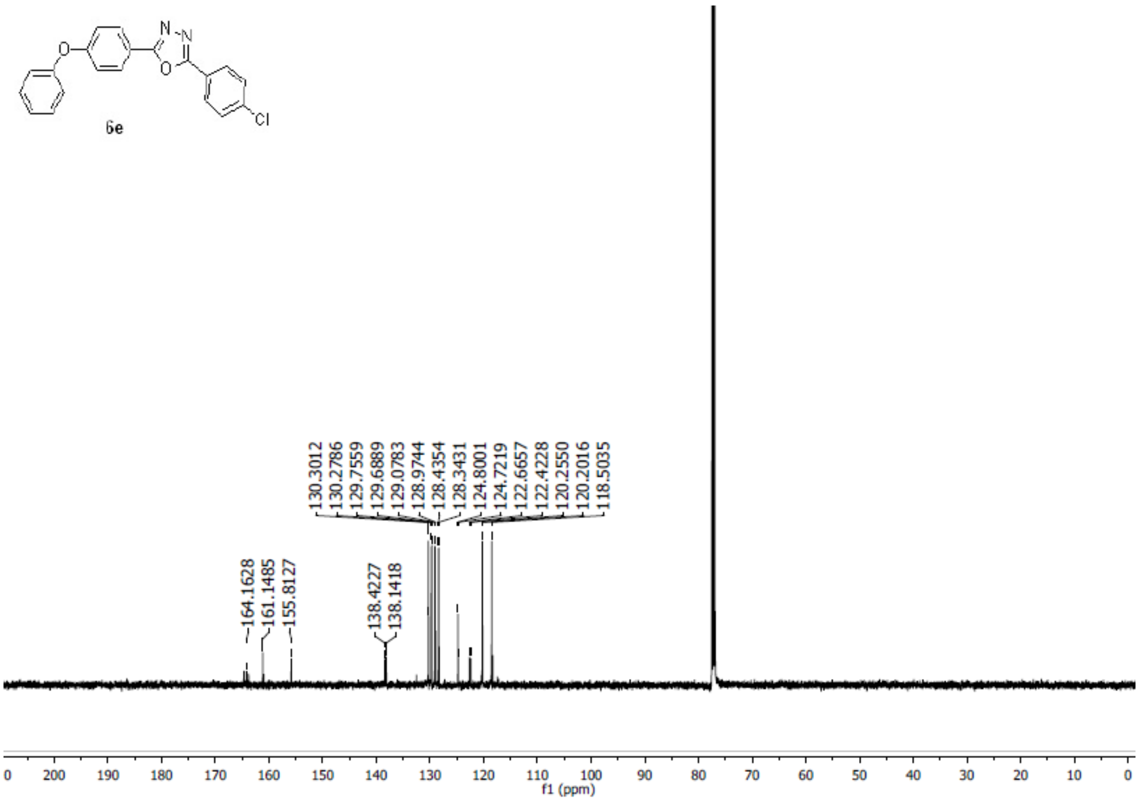

^{1}H NMR for compound 6f

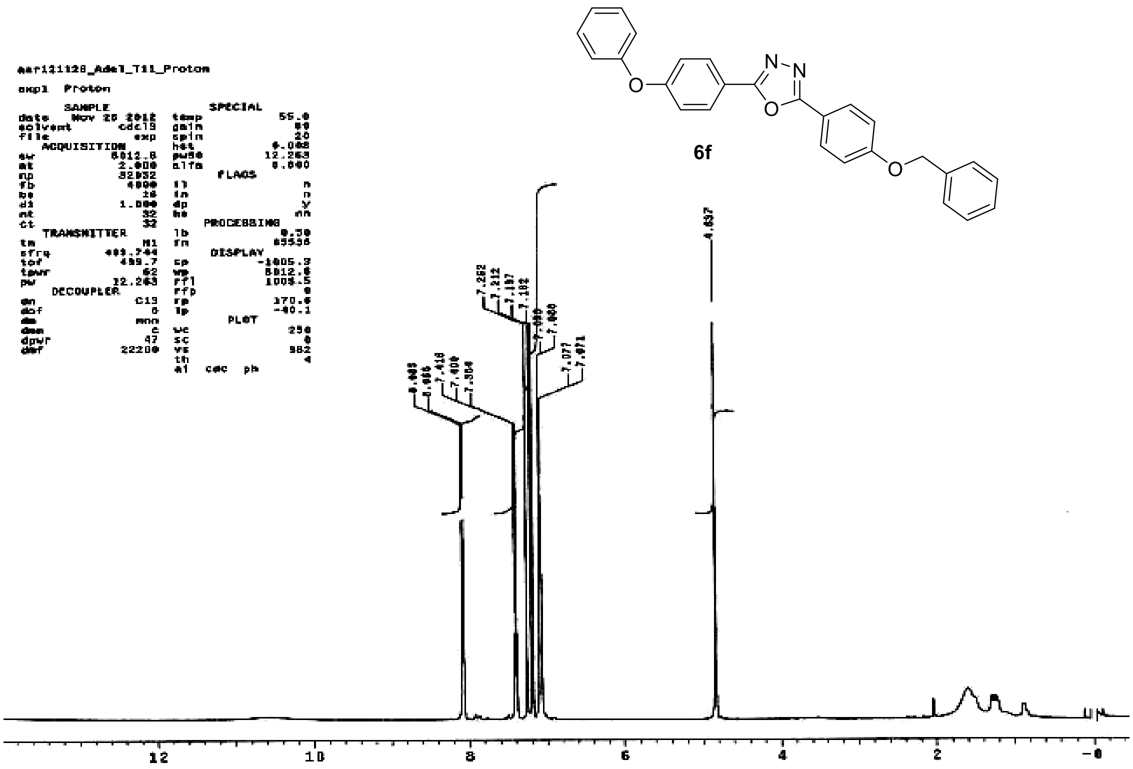

^{1}H NMR for compound 6g

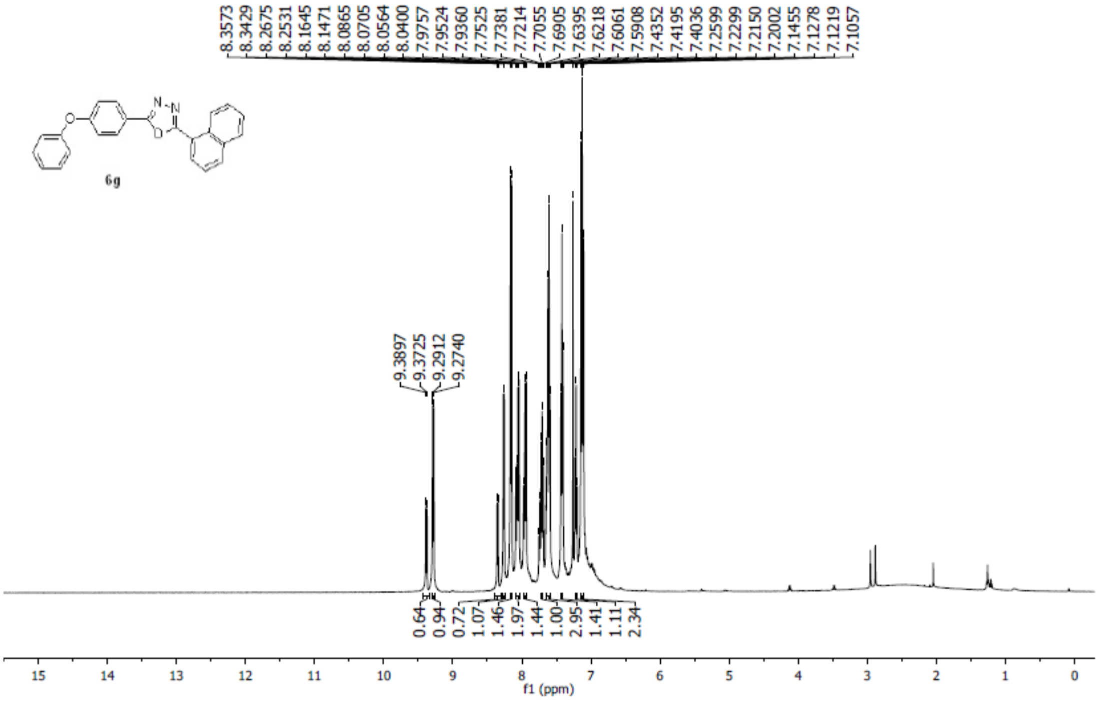

^{13}C NMR for compound 6g

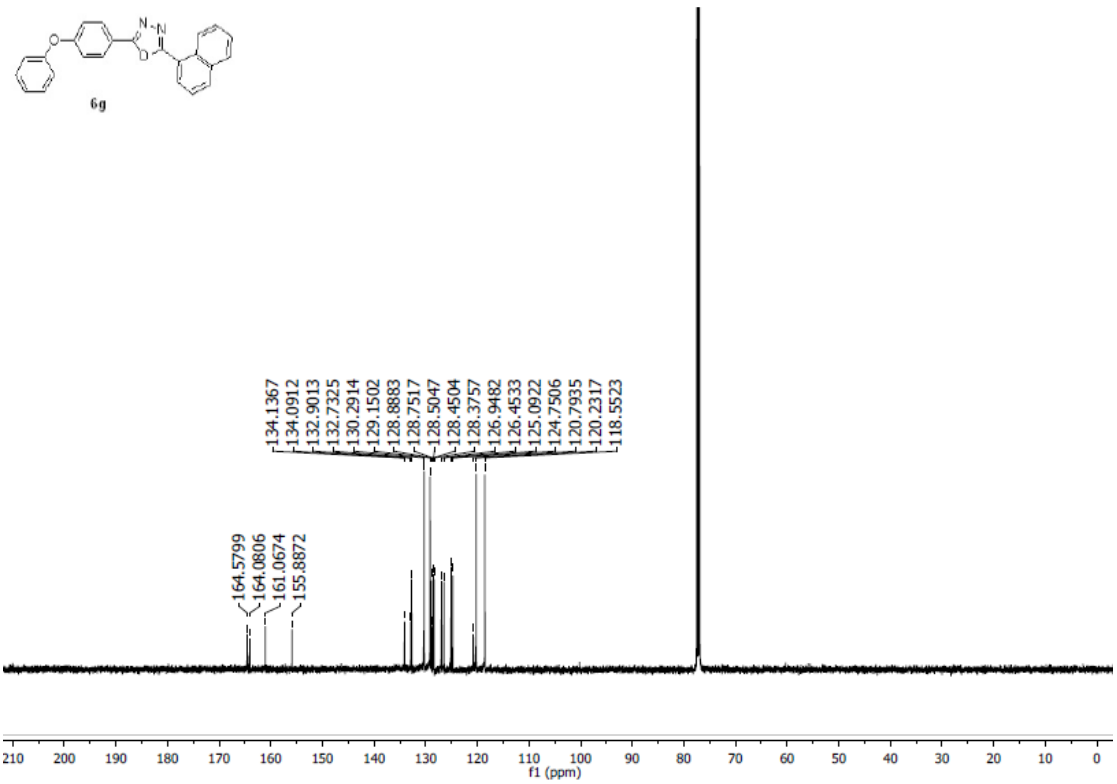

^{1}H NMR for compound 6h

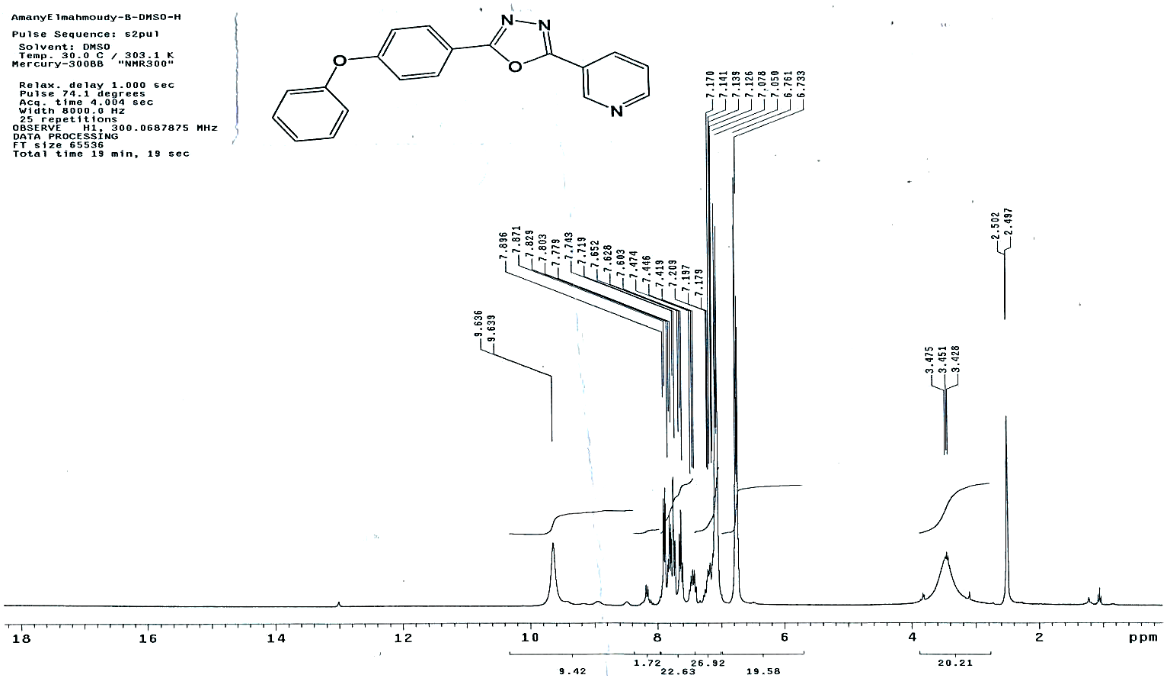

^{1}H NMR for compound 6i

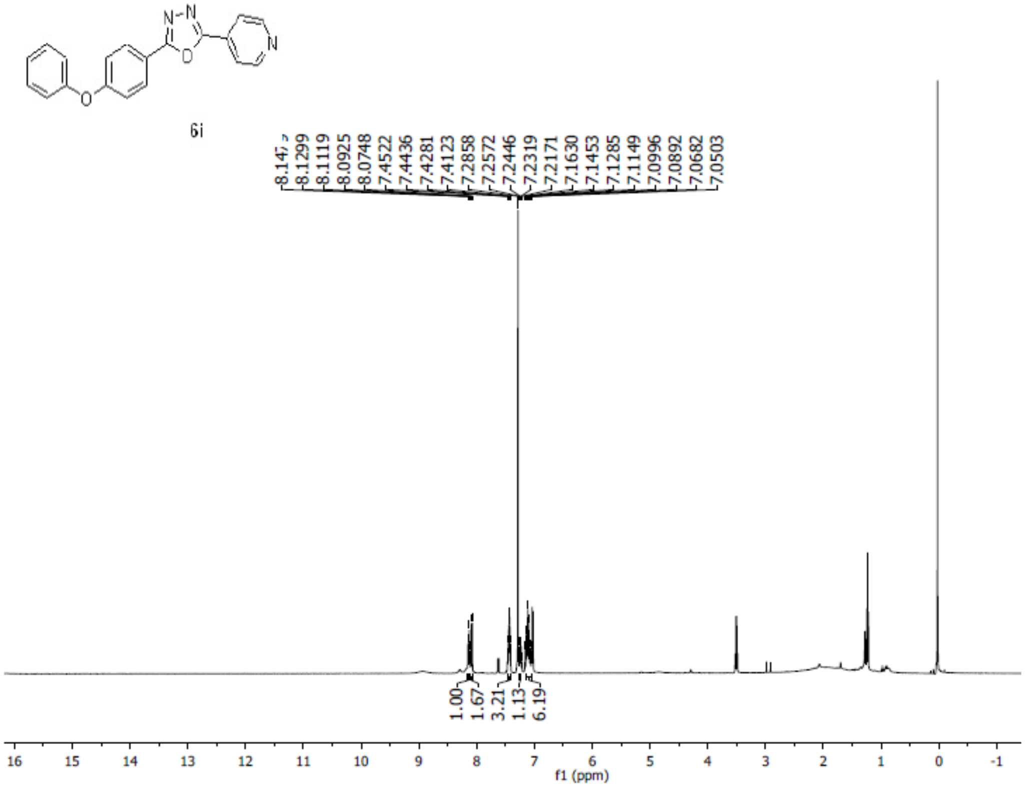

^{13}C NMR for compound 6i

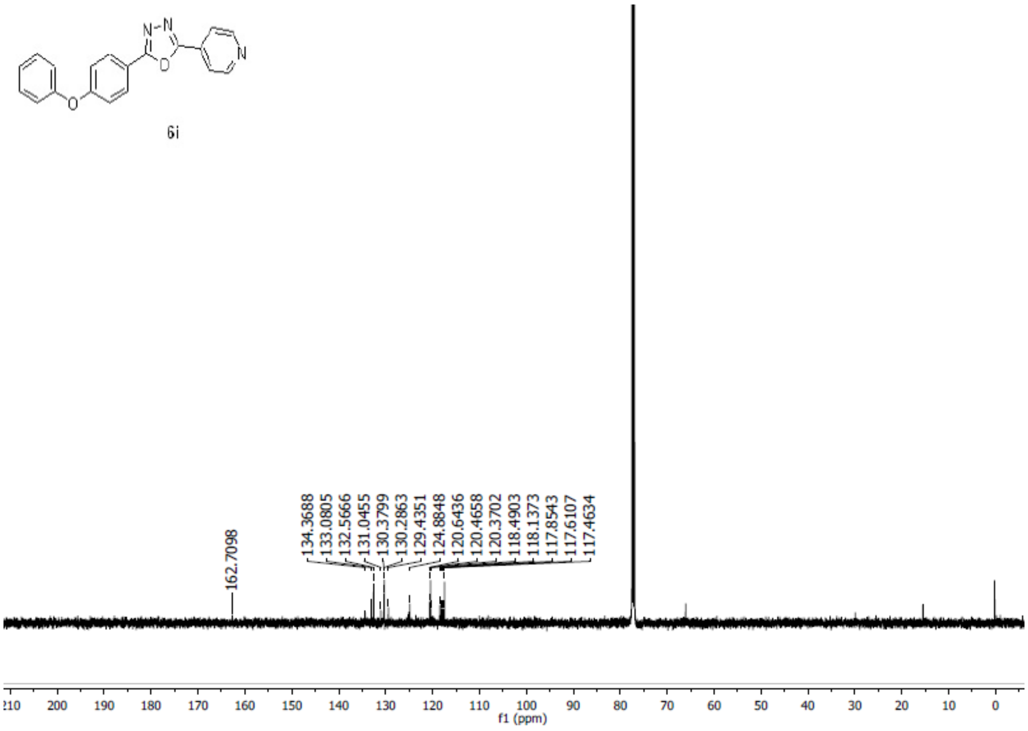

¹H NMR for compound 6j

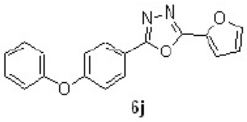

6j

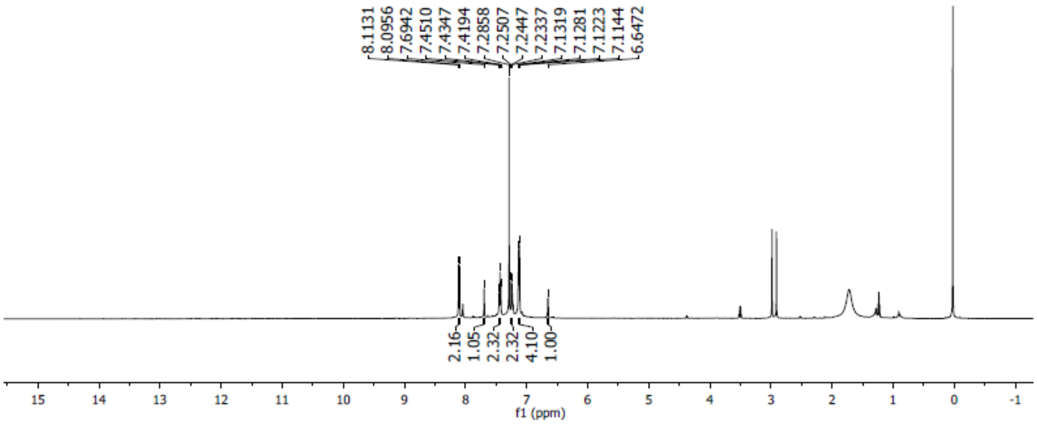

¹³C NMR for compound 6j

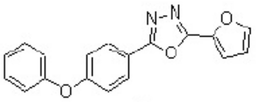

6j

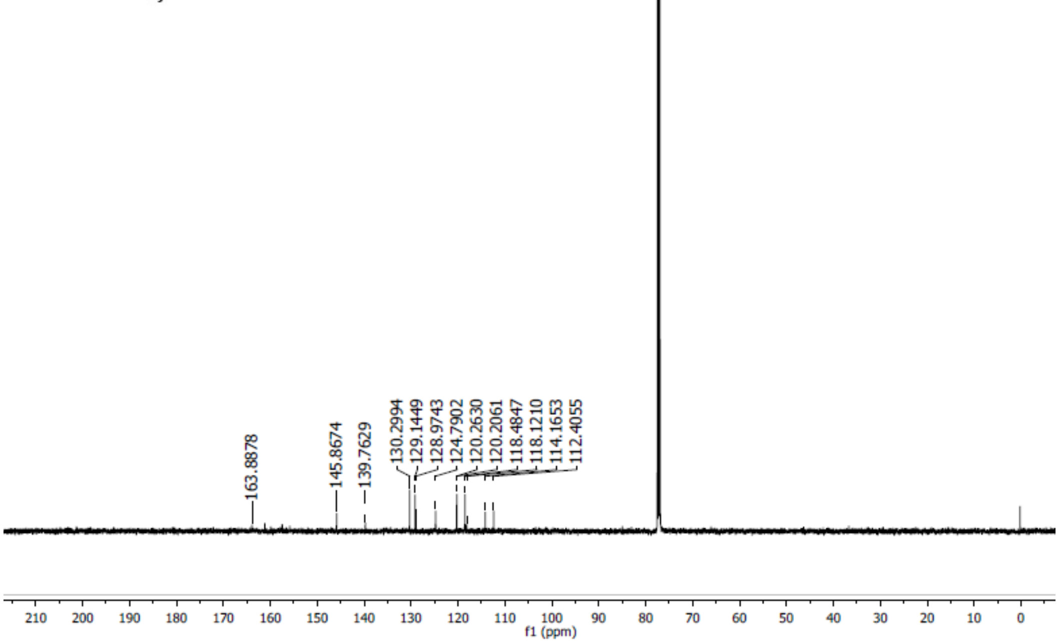

^{1}H NMR for compound 6k

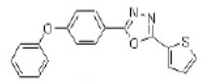

6k

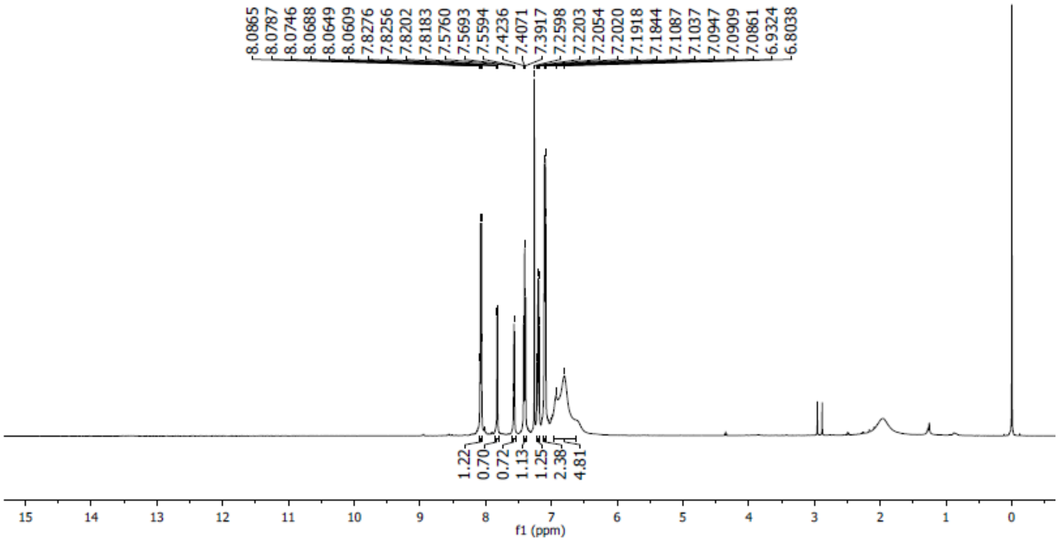

^{13}C NMR for compound 6k

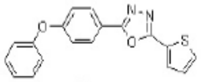

6k

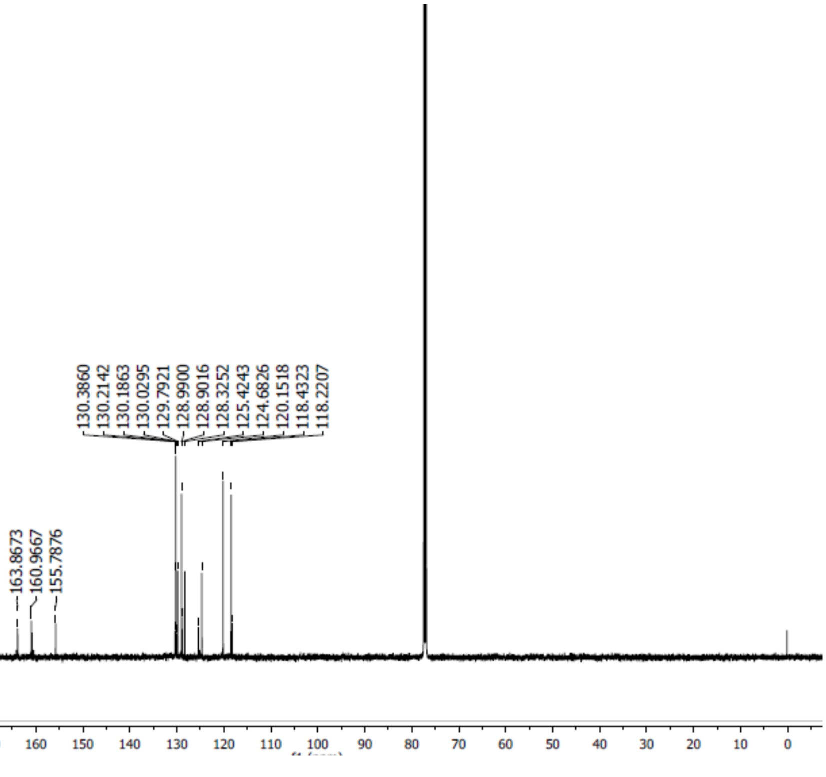

^{1}H NMR for compound 6l

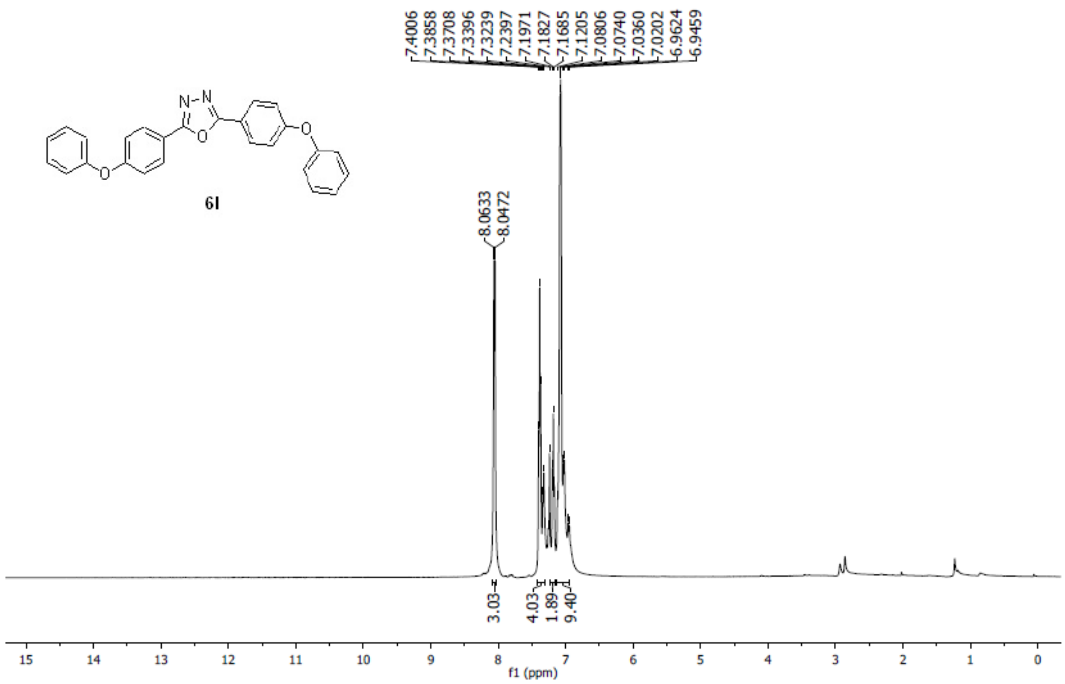

^{13}C NMR for compound 6l

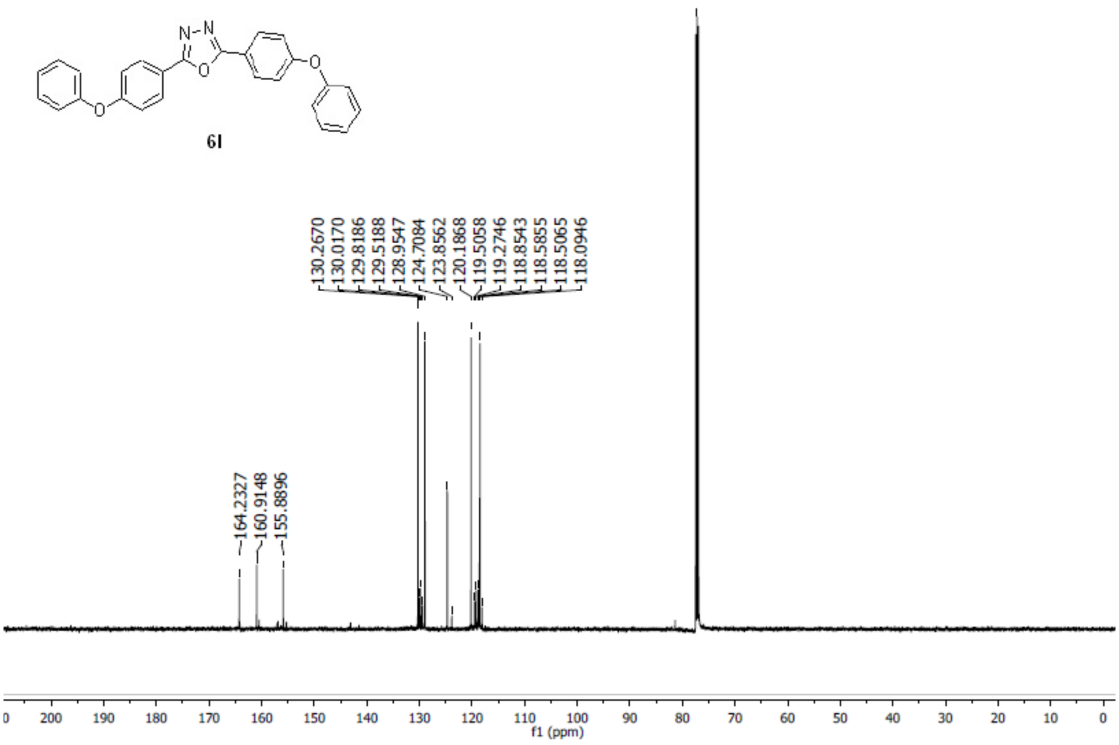

^{1}H NMR for compound 8a

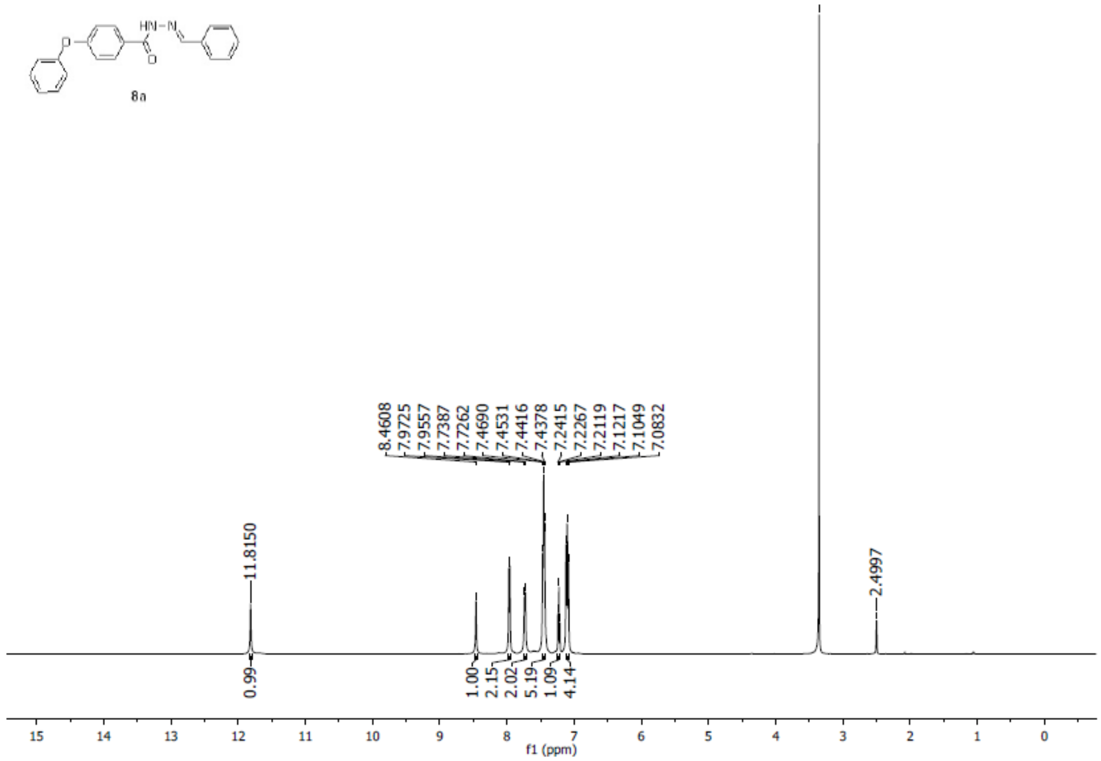

^{13}C NMR for compound 8a

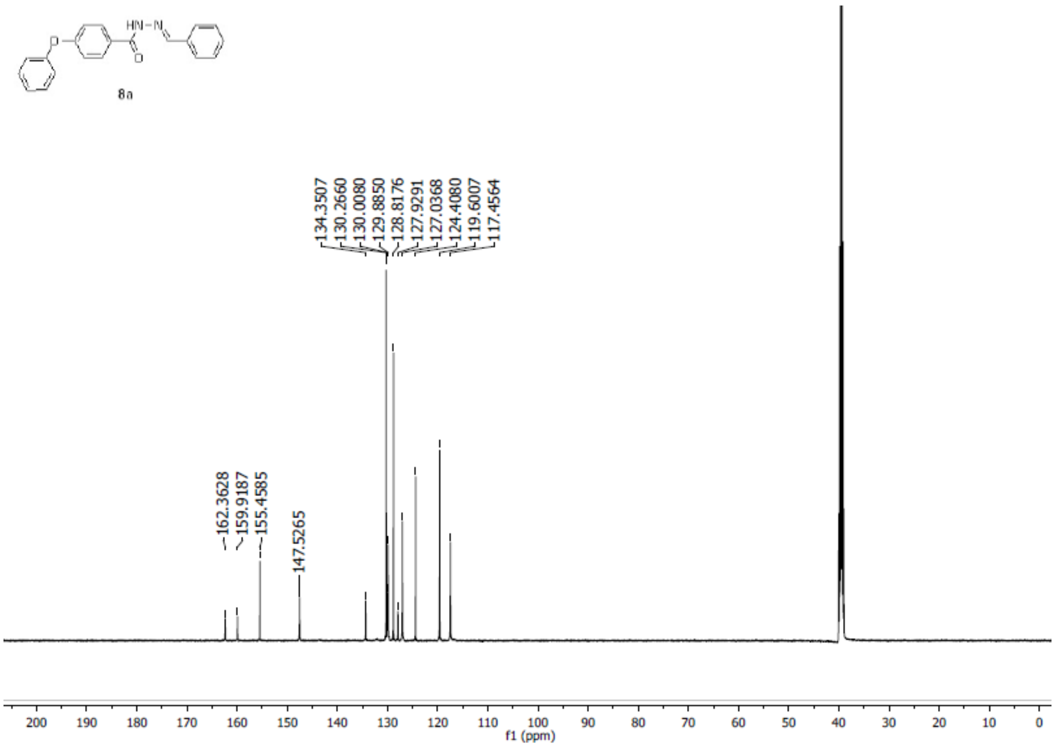

^{1}H NMR for compound 8b

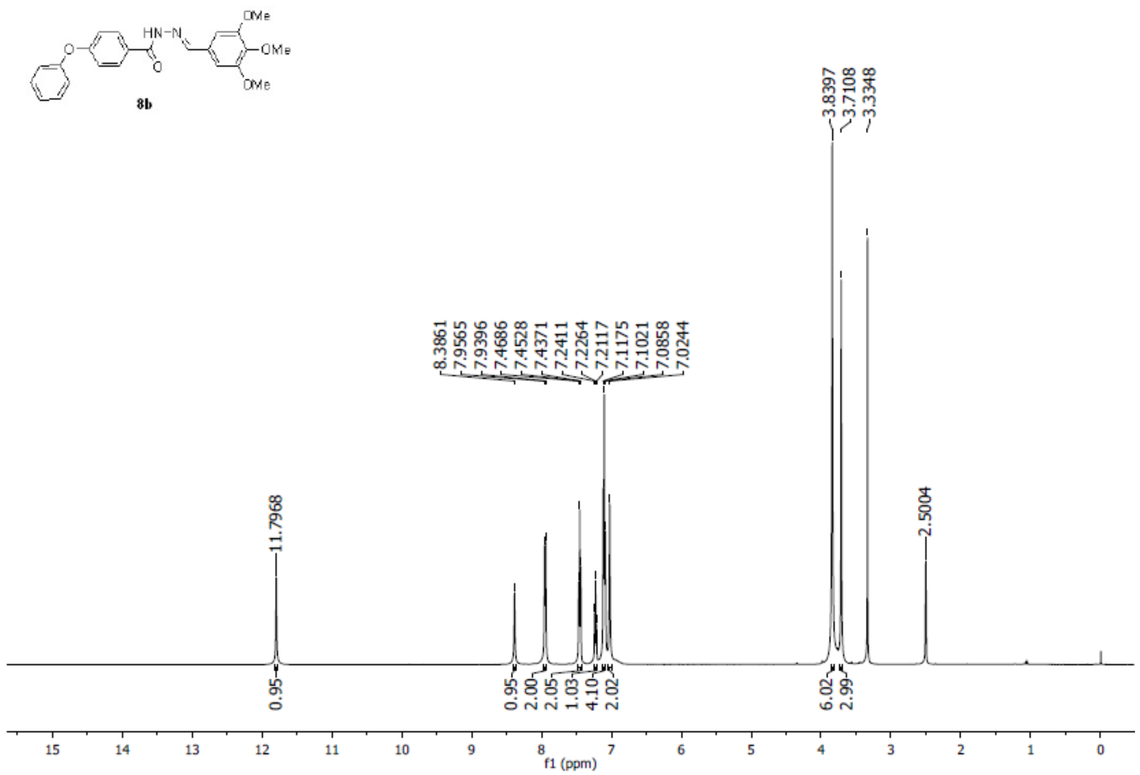

^{13}C NMR for compound 8b

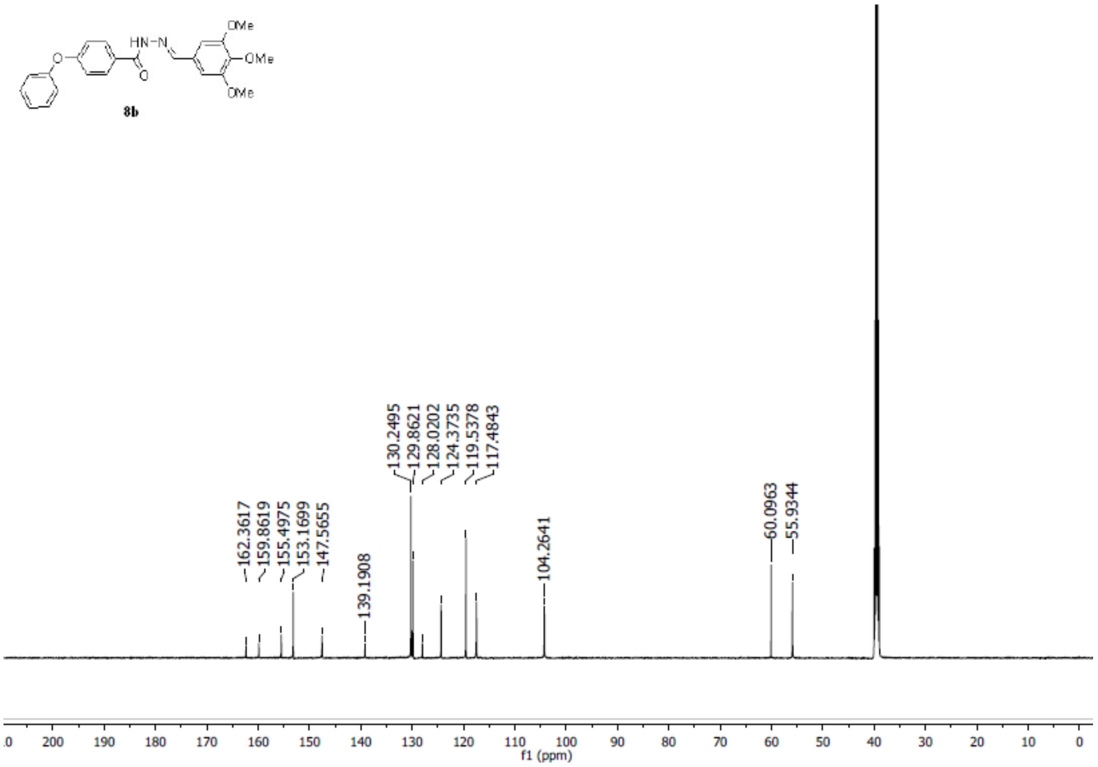

^{1}H NMR for compound 8c

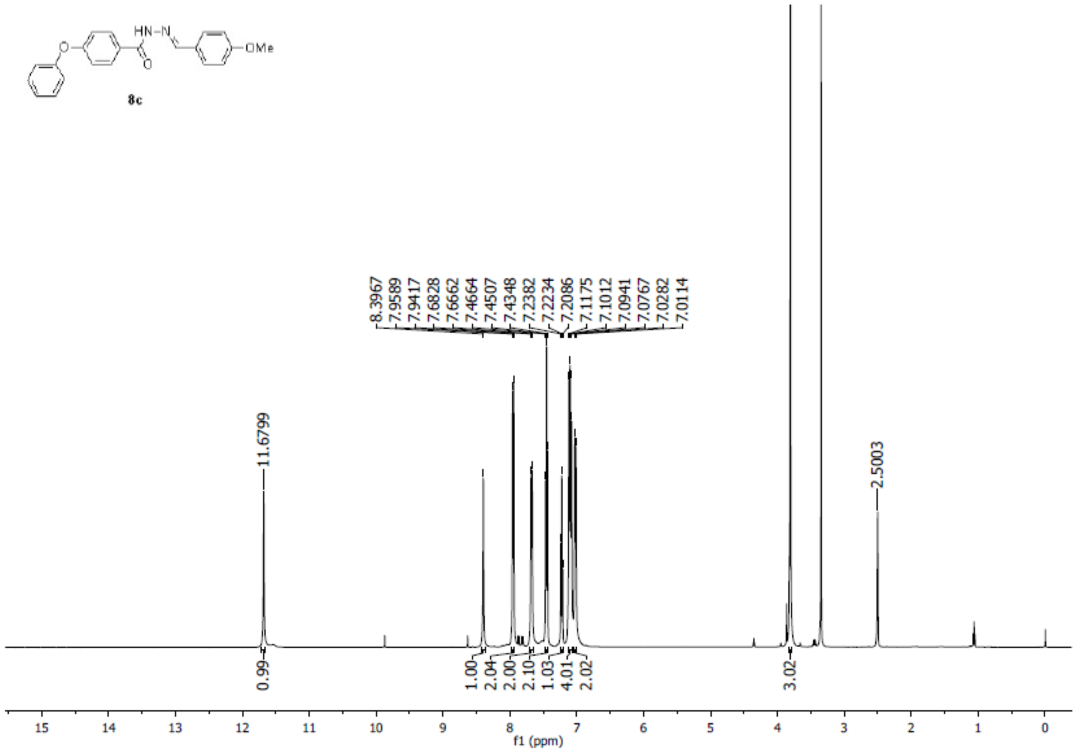

^{13}C NMR for compound 8c

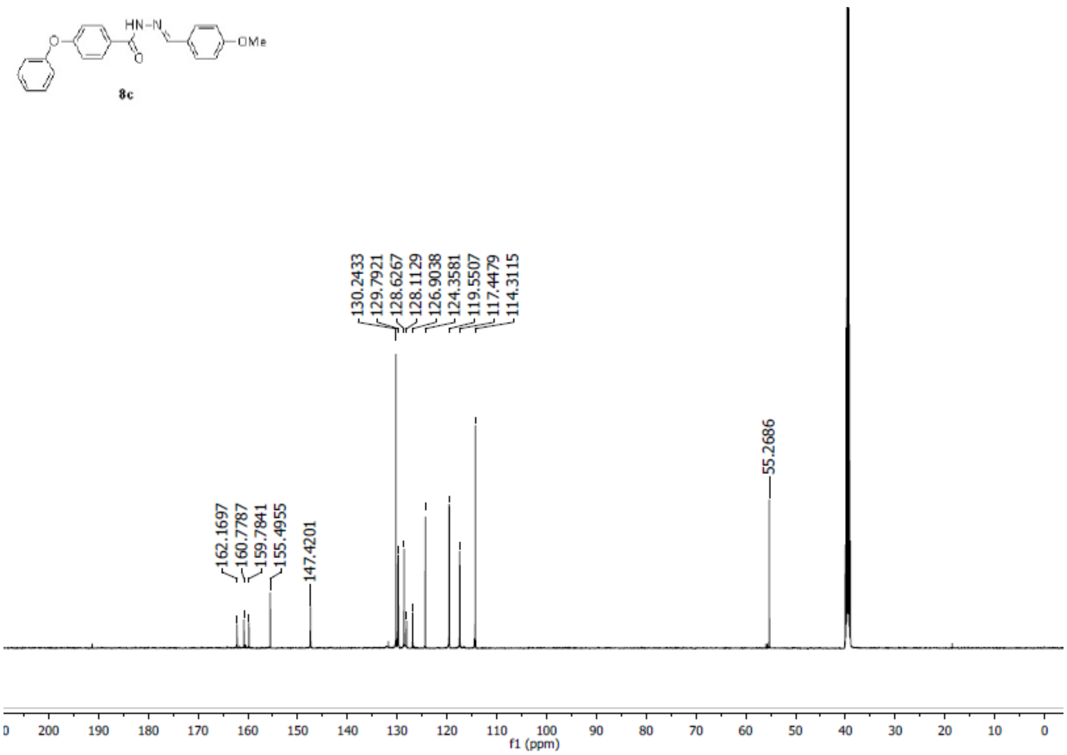

^{1}H NMR for compound 8d

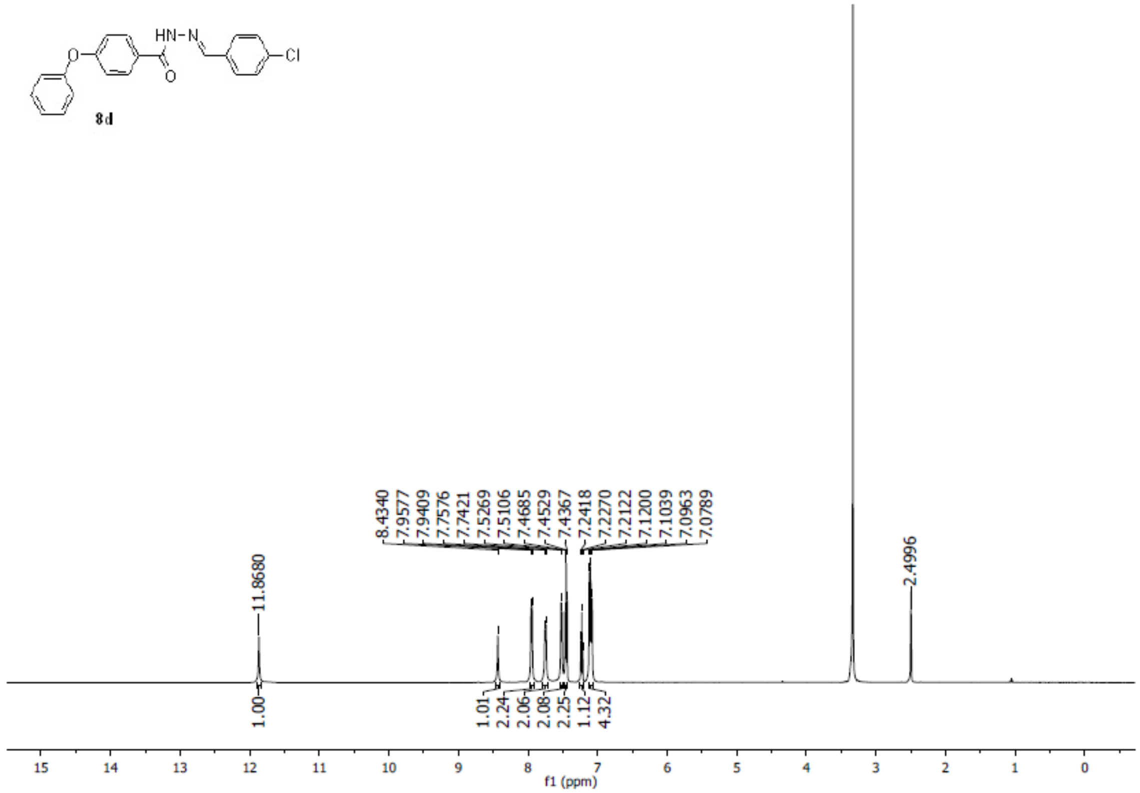

^{13}C NMR for compound 8d

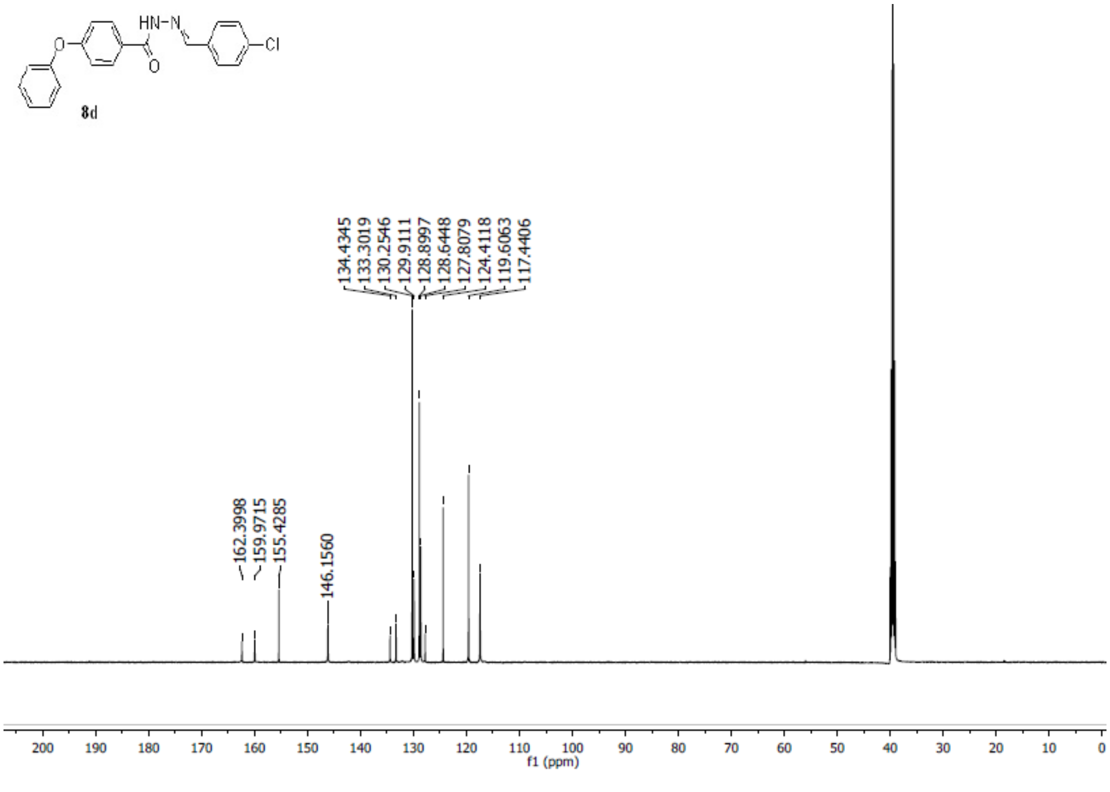

^{1}H NMR for compound 8e

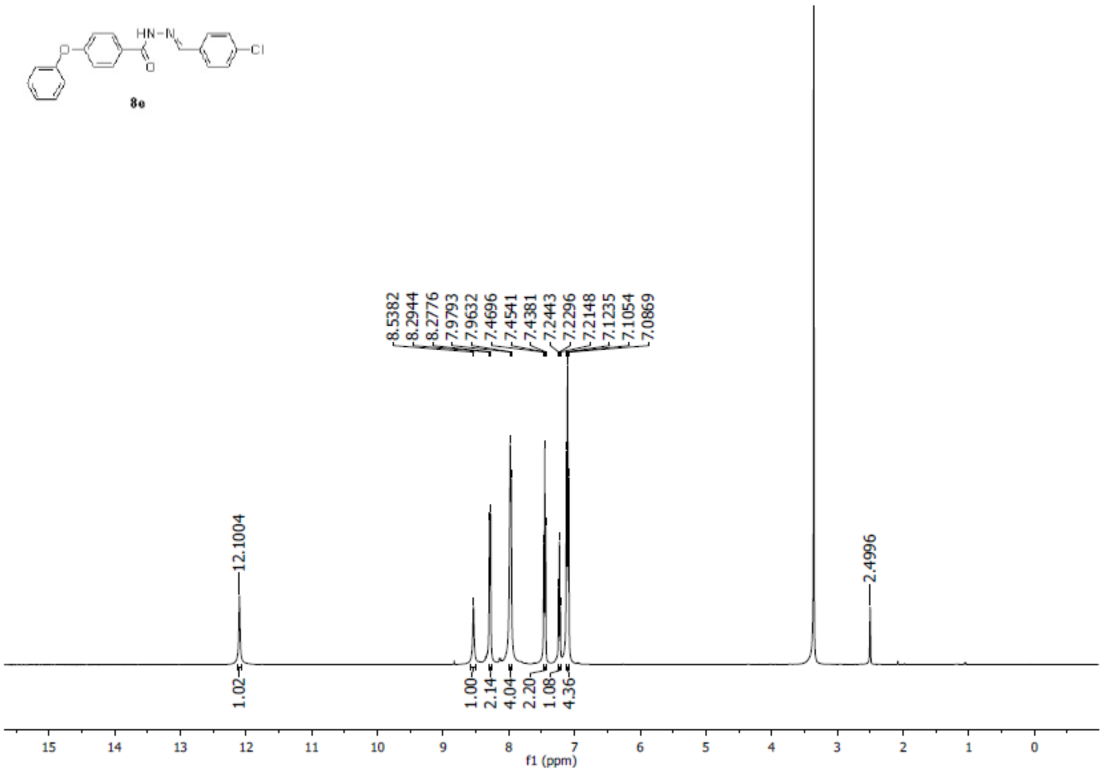

^{13}C NMR for compound 8e

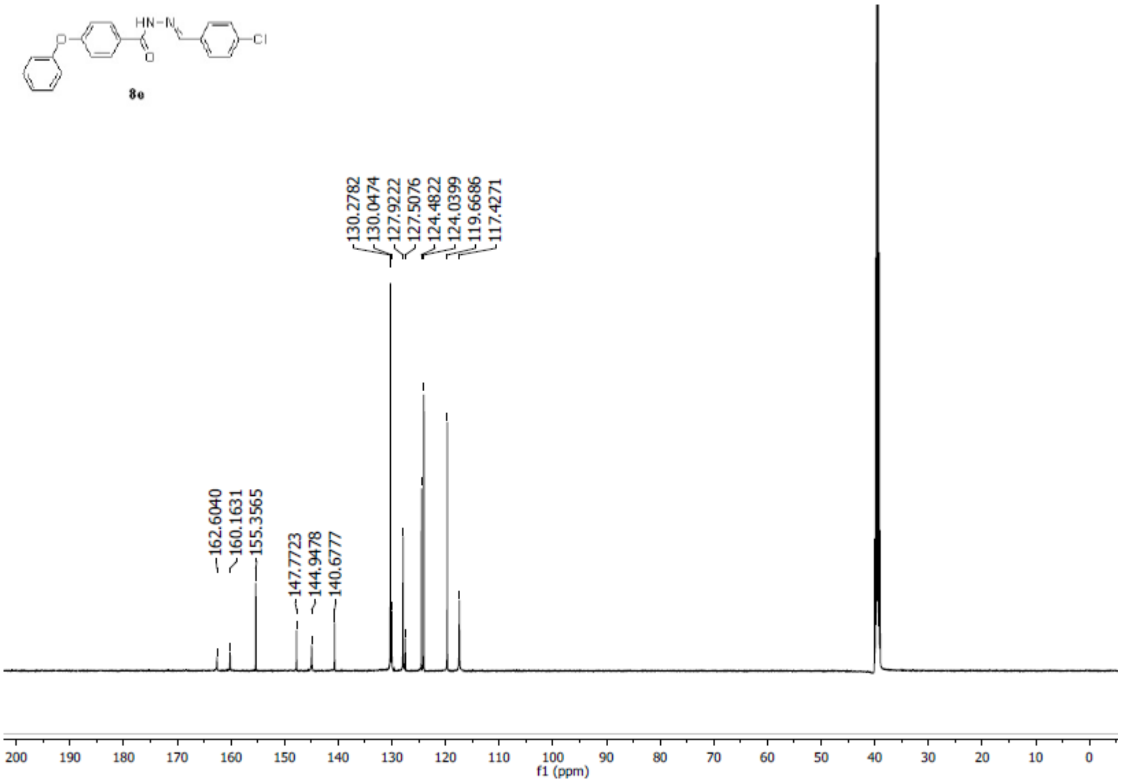

^{1}H NMR for compound 8f

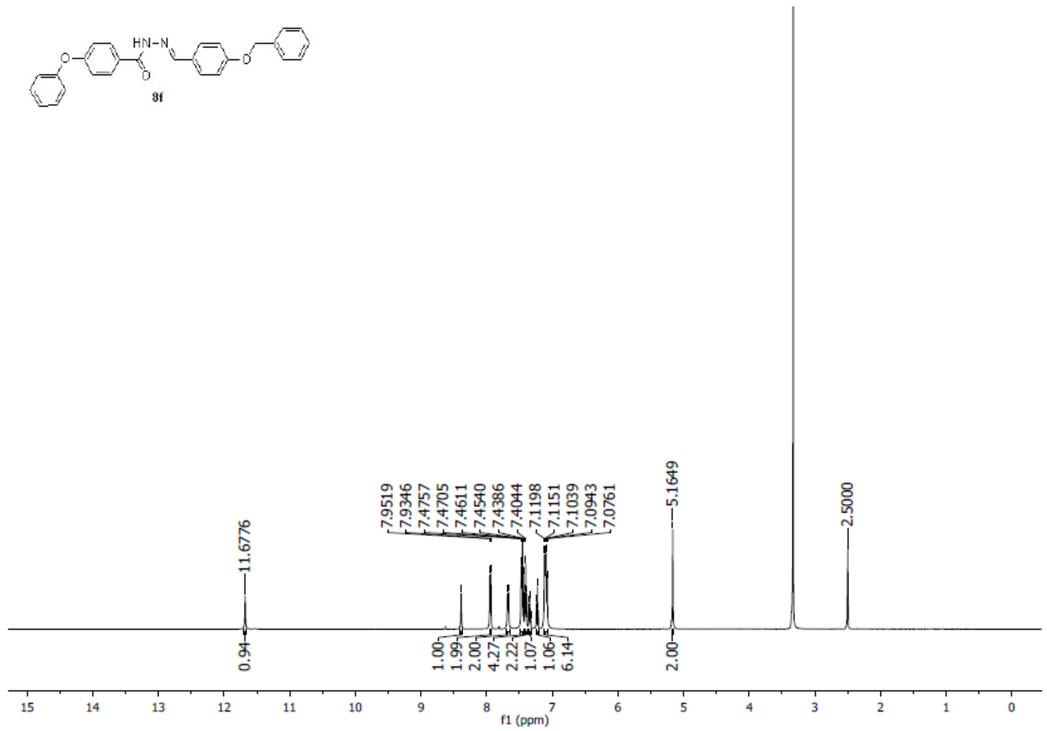

^{13}C NMR for compound 8f

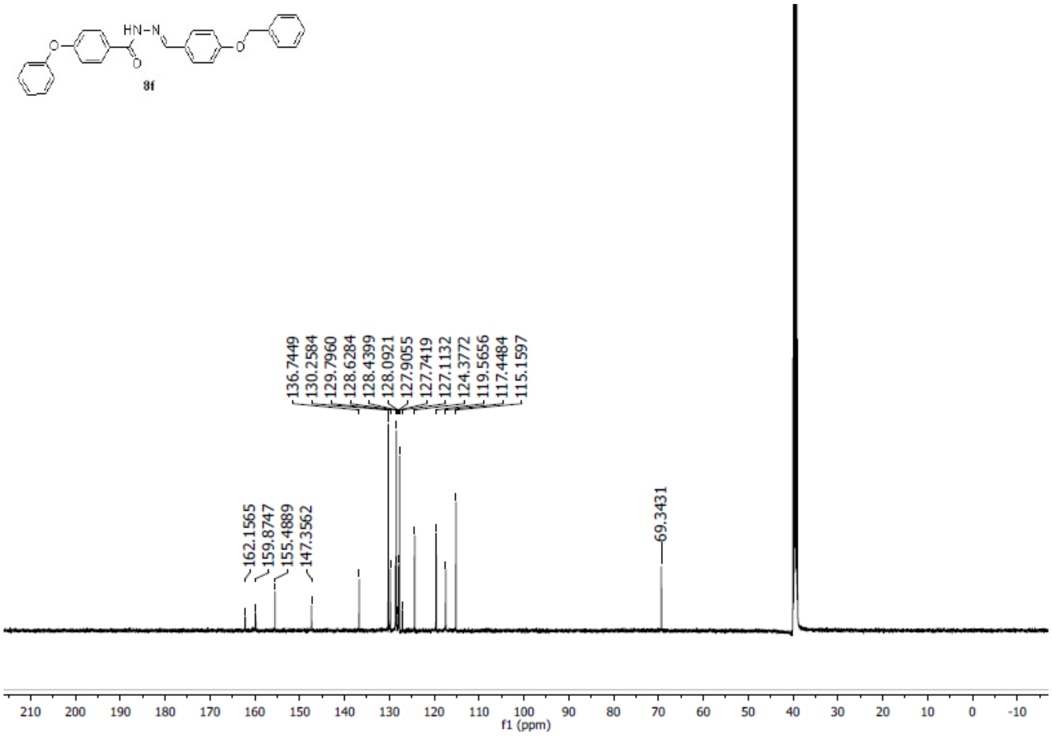

^{1}H NMR for compound 8g

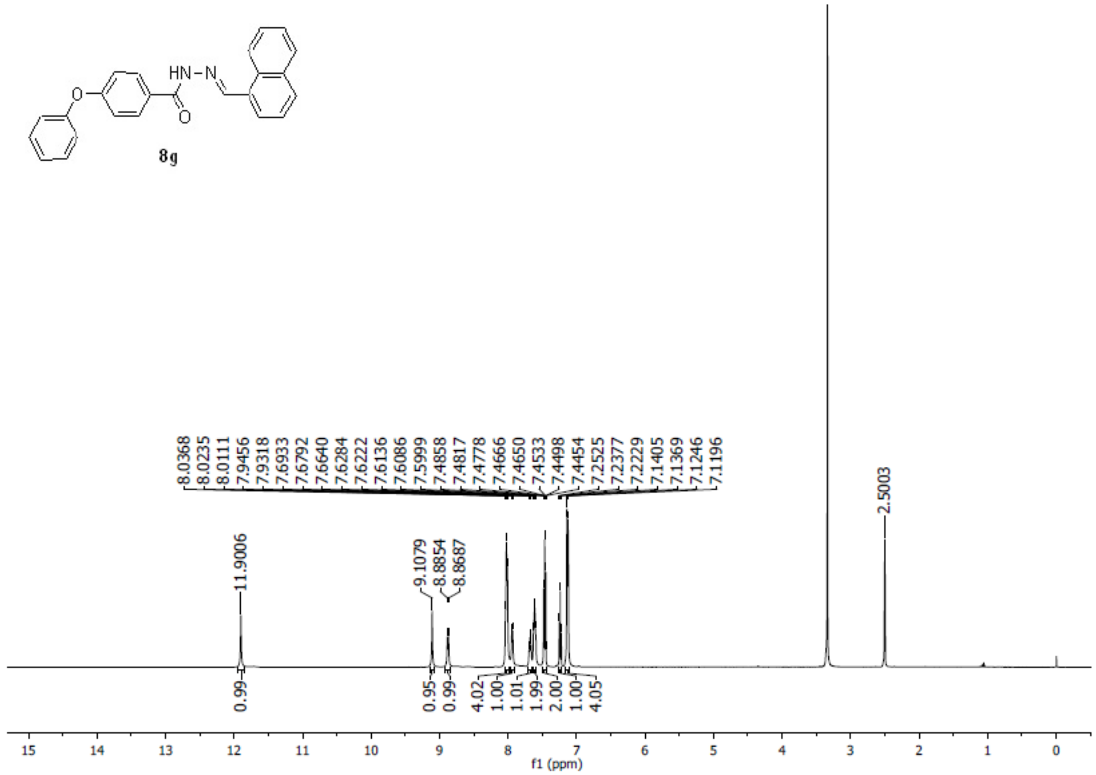

^{13}C NMR for compound 8g

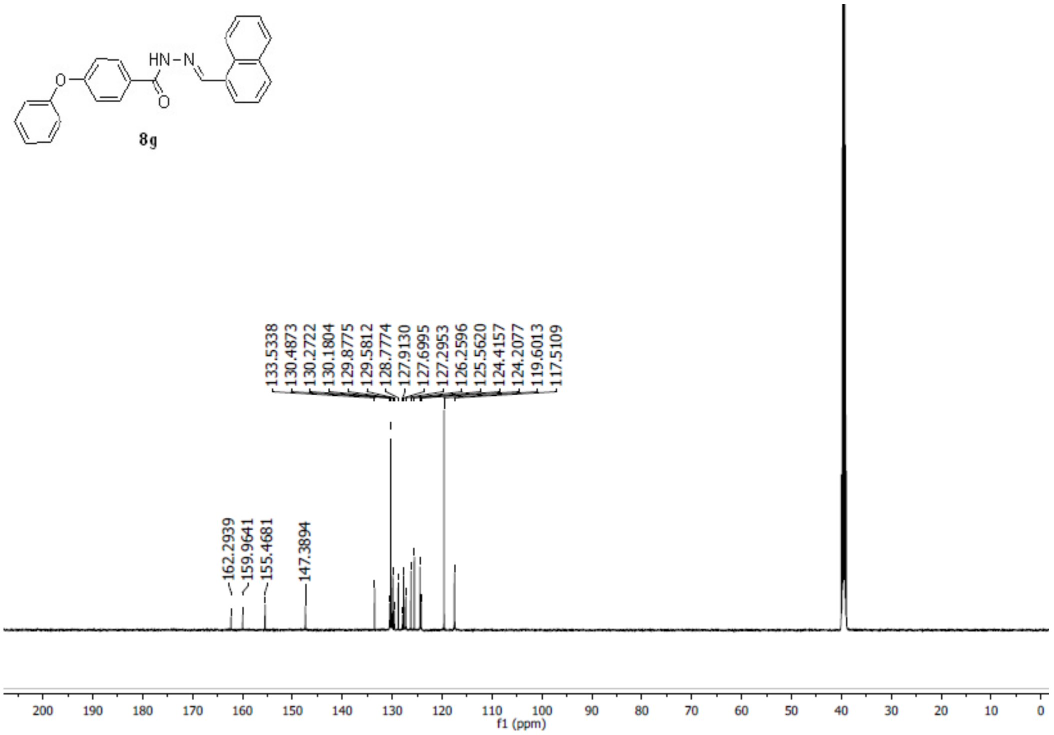

^{1}H NMR for compound 8h

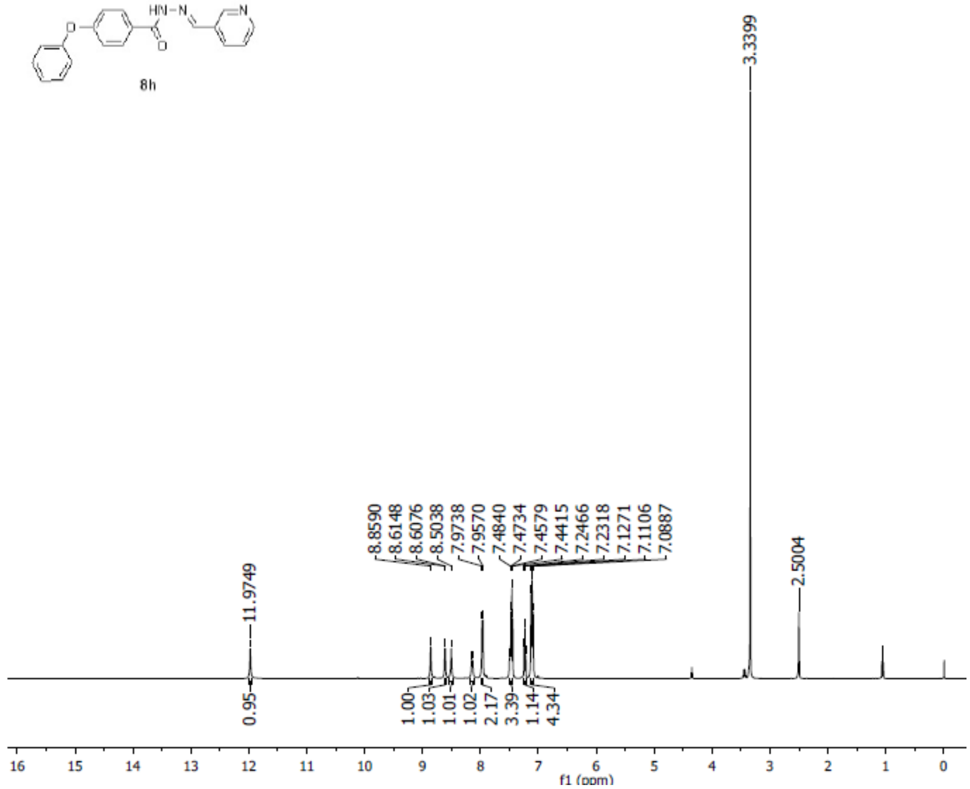

^{13}C NMR for compound 8h

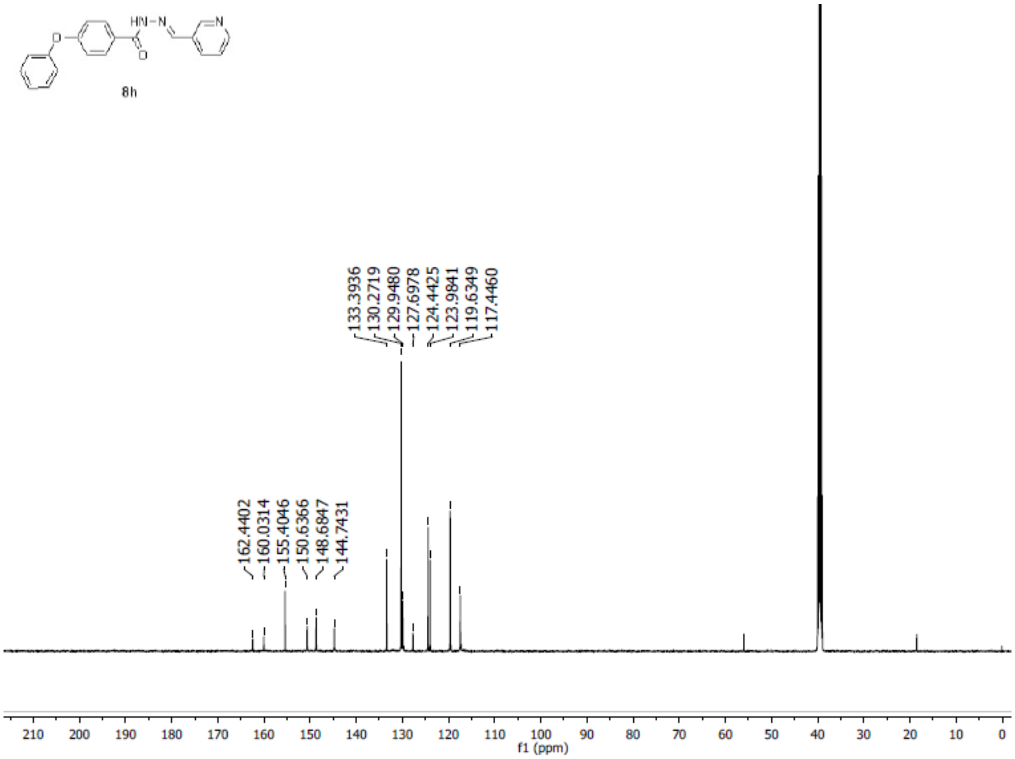

^{1}H NMR for compound 8i

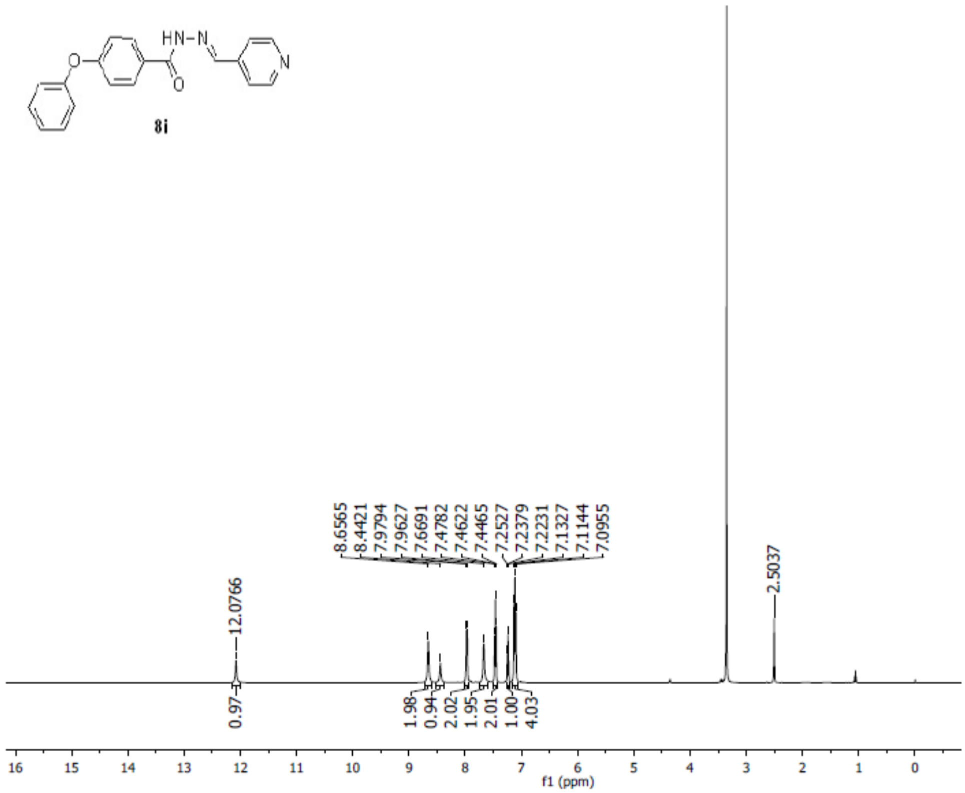

^{13}C NMR for compound 8i

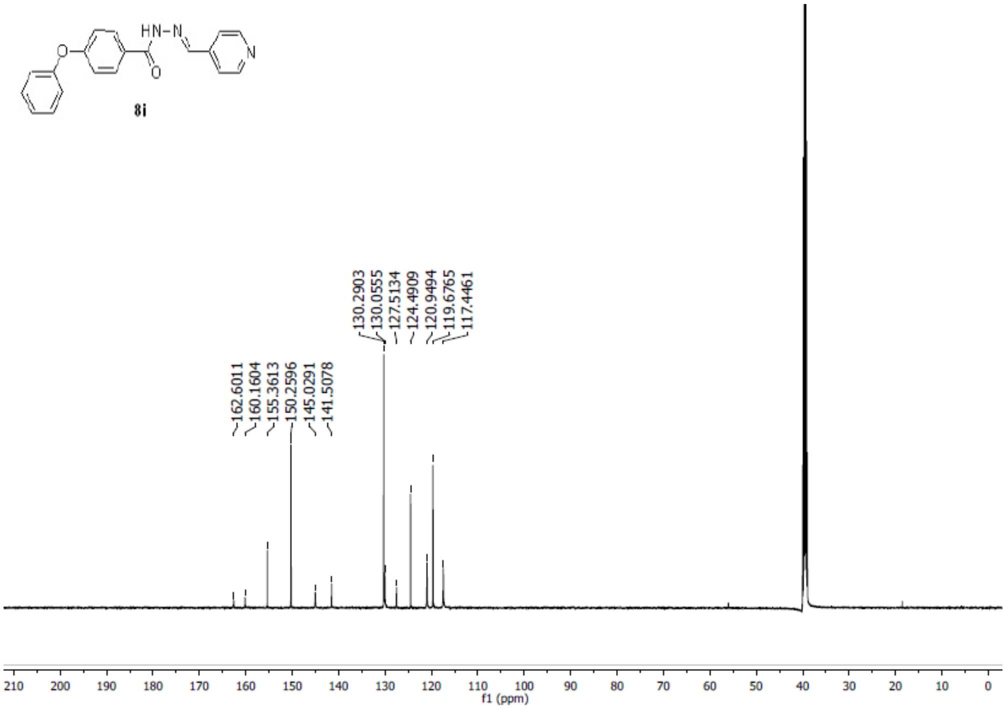

^{1}H NMR for compound 8j

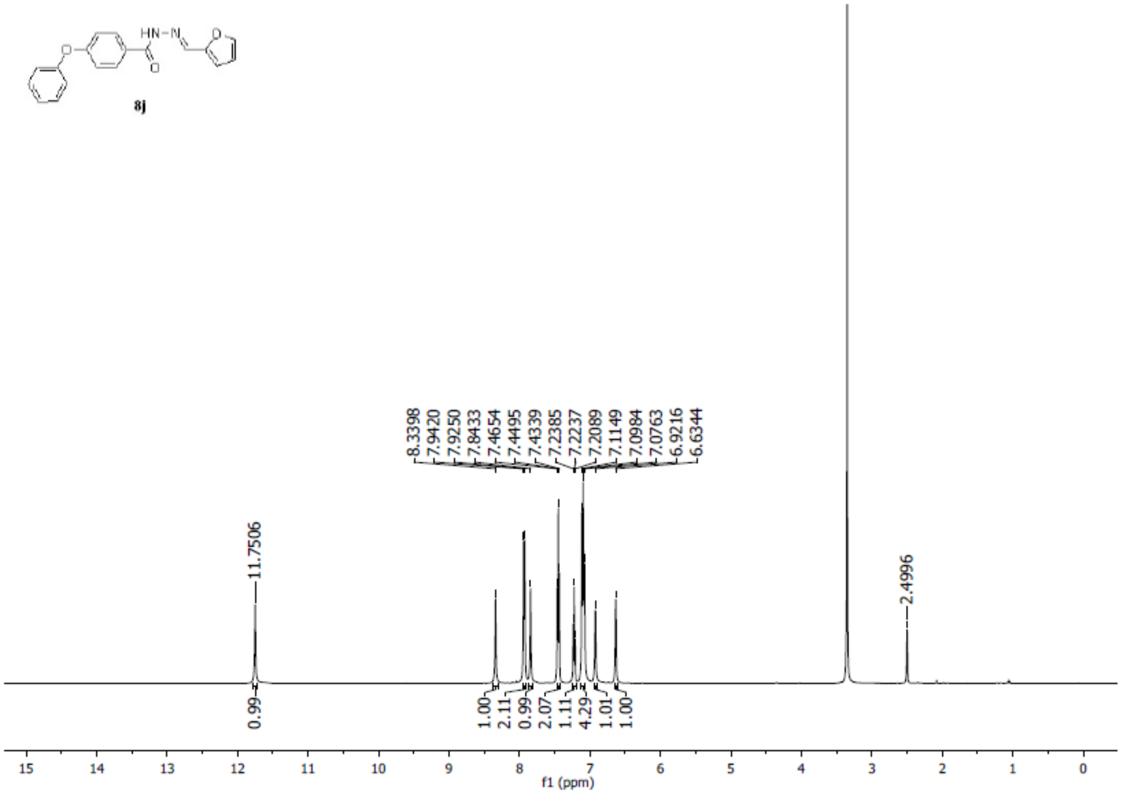

^{13}C NMR for compound 8j

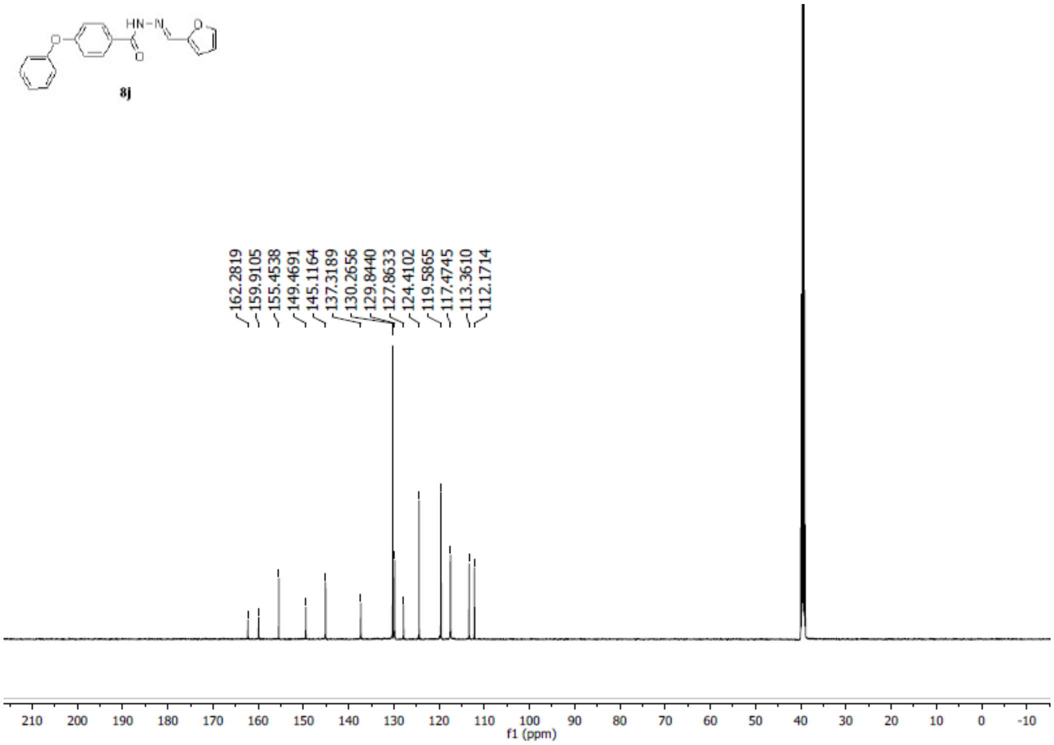

^{1}H NMR for compound 8k

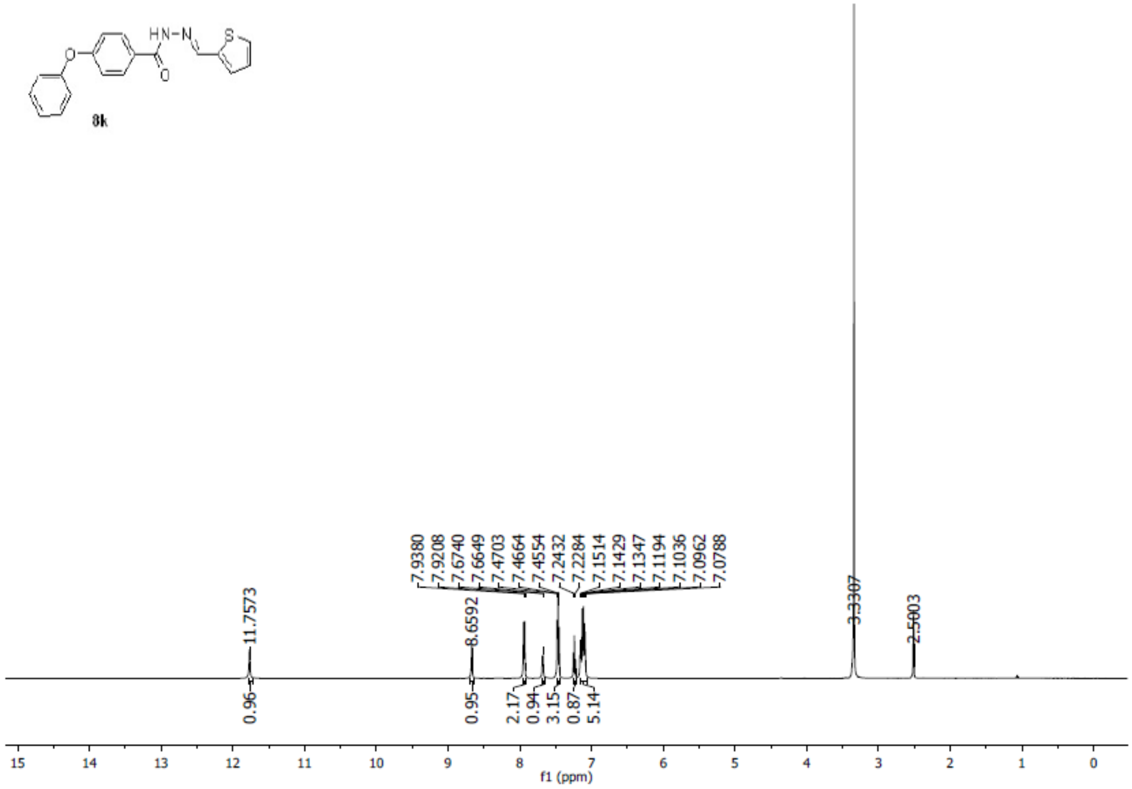

^{13}C NMR for compound 8k

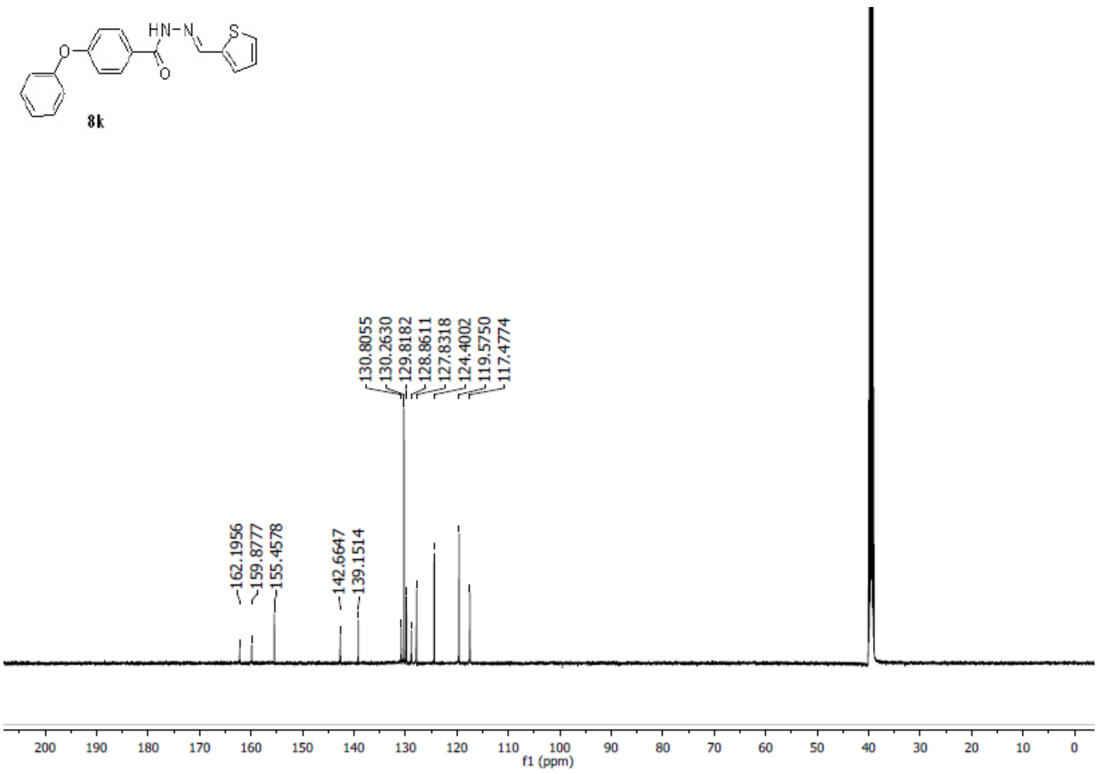

Supplement: Supplementary file 1 [file molecules-25-03125-s001.pdf]
